# Bacterial extracellular vesicle-coated multi-antigenic nanovaccines protect against drug-resistant *Staphylococcus aureus* infection by modulating antigen processing and presentation pathways

**DOI:** 10.7150/thno.44564

**Published:** 2020-05-30

**Authors:** Gang Chen, Yanan Bai, Zhenzhen Li, Fei Wang, Xuelian Fan, Xin Zhou

**Affiliations:** 1 College of Veterinary Medicine, Yangzhou University, Yangzhou 225009, China.; 2 Institute of Comparative Medicine, Yangzhou University, Yangzhou 225009, China.; 3 Jiangsu Co-innovation Center for Prevention and Control of Important Animal Infectious Diseases and Zoonoses, Yangzhou University, Yangzhou 225009, China.; 4 Joint International Research Laboratory of Agriculture and Agri-Product Safety, the Ministry of Education of China, Yangzhou University, Yangzhou 225009, China.

**Keywords:** bacterial extracellular vesicles, nanovaccines, drug-resistant *Staphylococcus aureus*, cross-presentation, CD8^+^ T cells

## Abstract

**Background:** Vaccination provides an alternative to antibiotics in addressing drug-resistant *Staphylococcus aureus* (*S. aureus*) infection. However, vaccine potency is often limited by a lack of antigenic breadth and a demand on the generation of antibody responses alone.

**Methods:** In this study, bacterial extracellular vesicles (EVs) coating indocyanine green (ICG)-loaded magnetic mesoporous silica nanoparticles (MSN) were constructed as multi-antigenic vaccines (EV/ICG/MSN) with the ability to modulate antigen presentation pathways in dendritic cells (DCs) to induce cellular immune responses.

**Results:** Exposing the EV/ICG/MSNs to a laser could promote DC maturation and enhance the proteasome-dependent antigen presentation pathway by facilitating endolysosomal escape, improving proteasome activity, and elevating MHC-I expression. Immunization by EV/ICG/MSNs with laser irradiation *in vivo* triggered improved CD8^+^ T cell responses while maintaining CD4^+^ T cell responses and humoral immunity. In addition, *in vivo* tracking data revealed that the vaccine could be efficiently transported from the injection site into lymph nodes. Skin infection experiments showed that the vaccine not only prevented and treated superficial infection but also decreased bacterial invasiveness, thus strongly suggesting that EV/ICG/MSNs were effective in preventing complications resulting from the introduction of *S. aureus* infections.

**Conclusion:** This multi-antigenic nanovaccine-based modulation of antigen presentation pathways provides an effective strategy against drug-resistant *S. aureus* infection.

## Introduction

*Staphylococcus aureus* (*S. aureus*), a major human pathogen, is known to acquire resistance to a variety of antibiotics 1. For instance, methicillin-resistant *S. aureus* (MRSA) strains have spread globally 2. Once the human body is infected by drug-resistant *S. aureus*, these strains cannot be completely removed by antibiotics, even at high doses 3,4. *S. aureus* colonizes the skin and mucosal membranes and penetrates into deeper tissue frequently, leading to skin infections and systemic infections of the heart, bones, and lungs 5. Endocarditis, sepsis, and toxic shock syndrome are examples of fatal diseases caused by *S. aureus* 2. The World Health Organization recently reported that *S. aureus* is a serious opportunistic pathogen that parasitizes one-third of the healthy human population and is the leading cause of bacterial infections in the world 6. However, slow progress in the development of new antibiotics has prompted researchers to search for novel approaches to deal with drug-resistant *S. aureus* infections.

Effective vaccination provides a viable alternative to antibiotics, which is believed to be simpler and better than traditional treatment of drug-resistant *S. aureus* infections 7. Vaccines prevent infectious diseases by training the host immune system to recognize pathogen-associated antigens 8. Most studies over the years have focused on vaccine-induced antibody production because antibody responses to *S. aureus* play an important role in blocking toxins 9,10. However, two Phase III clinical trials only pinning hopes on antibody responses failed to protect patients against *S. aureus* infections 11,12. The employment of single or double antigens and the demand for the generation of an antibody response alone by the immune system may limit the development of *S. aureus* vaccines 9.

Recent studies have verified the requirement for a robust T cell immune response to improve efficacy in addressing *S. aureus* infections 13-15. Cytotoxic CD8^+^ T lymphocytes (CTL) are the primary cells that eliminate intracellular pathogens 16,17. *S. aureus* is able to invade and survive inside host cells, and this characteristic is associated with chronic or recurrent infections and results in subsequent complications 18. Therefore, CD8^+^ T cells are currently at the forefront of *S. aureus* vaccine development in this emerging field of intracellular *S. aureus* 19,20. CD4^+^ T cells, another important T cell type, are not effective against intracellular bacteria. The cytokines secreted by CD4^+^ T cells have only indirect effects on the survival of intracellular pathogens. However, they still play important roles in stimulating the production of antibodies and generating primary immune responses or maintaining memory CTL 21. Antigen presenting cells (APCs), in particular dendritic cells (DCs), are essential for initiating and regulating CD4^+^ and CD8^+^ T cell immune responses 22. Antigen presentation pathways within DCs determine the type of cellular immune responses. The lysosome-dependent pathway in DCs is the most common fate for exogenous antigens, which are degraded into peptides and loaded onto MHC-II for presentation to CD4^+^ helper T cells 23. In contrast, to activate CD8^+^ T cells and elicit robust CTL responses, the antigens should localize to the cytosol and are presented to MHC-I through the proteasome-dependent pathway, a process called cross-presentation 2. One goal of modern vaccine design is to maintain the lysosome-dependent pathway and promote the proteasome-dependent pathway in the cytosol based on MHC-I antigen cross-presentation.

**Scheme 1:**
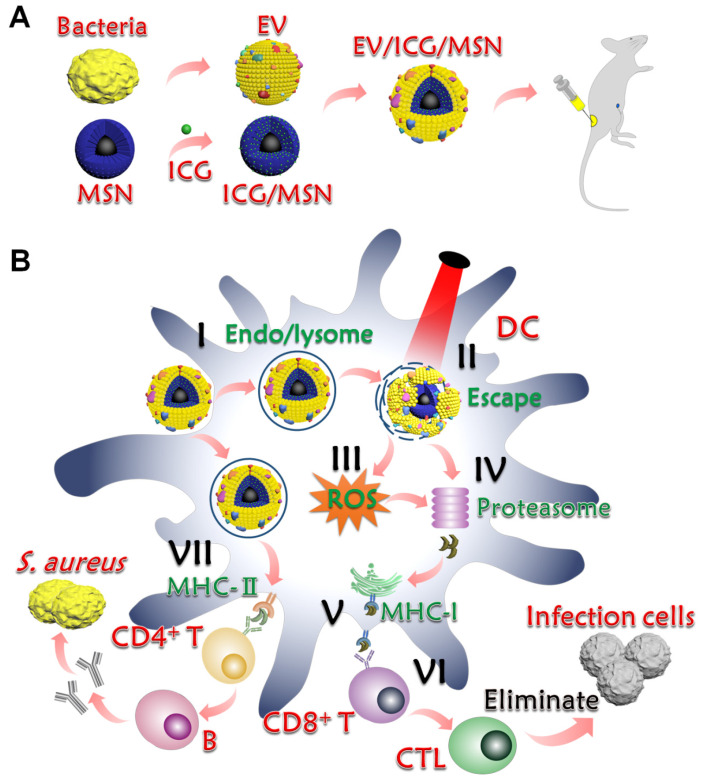
** Preparation and mechanism of action of EV-coated multi-antigenic nanovaccines. (A)** Preparation of EVs from *S. aureus*, ICG-loaded MSN (ICG/MSN) and EV-coated ICG/MSN (EV/ICG/MSN). **(B)** Proposed mechanism of action of EV/ICG/MSN nanovaccines. (I) Cellular uptake. (II) Photothermally triggered rupture of the endolysosomal membrane, dissociation of EV, and release of antigens into the cytoplasm for cross presentation. (III) ROS production triggered by endolysosome rupture enhances proteasome activity and downstream MHC-I antigen presentation. (IV) Antigens digested by the proteasome are subsequently bound by MHC-I-binding peptides. (V) MHC-I antigen presentation to CD8^+^ T cell. (VI) Activation of CD8^+^ T cells, followed by proliferation and differentiation into CTLs for clearing bacterial infection. (VII) Some of the antigens that remain in the endolysosome are presented by MHC-II to CD4^+^ T cells to further activate B cells.

Bacterial extracellular vesicles (EVs), mainly produced by Gram-negative bacteria, are spherical and bilayered nanovesicles 24. Secreted from the bacterial membrane, various EVs share a great similarity in their composition with their parent cells and include multiple immune stimulatory molecules, such as proteins, lipids, nucleic acids, and polysaccharides 25. Recent studies have revealed that Gram-positive *S. aureus* releases EVs 26. To avoid the limited effects of single or double antigens, we chose EVs as multi-antigenic vaccines and attempted to modulate antigen presentation pathways to effectively activate T cells responses (Scheme 1). In this study, we hypothesized that these nanovaccines could activate proteasome-dependent pathways following rupture of the endolysosome, delivering antigens into cytoplasm. The ROS production triggered by endolysosome rupture would enhance proteasome activity and downstream MHC-I antigen presentation. Parts of the antigens that remained in the endolysosome could be presented to MHC-II to activate CD4^+^ T cells for further activation of CD8^+^ T and B cells. To test this hypothesis, EVs secreted from drug-resistant *S. aureus* were coated on the surface of indocyanine green (ICG)-loaded magnetic mesoporous silica nanoparticles (MSNs), which represents a promising candidate for materials-based immunotherapy 27. The encapsulation of ICG aims to induce lysosome escape by laser irradiation because ICG molecules can absorb photons to produce heat that breaks apart the endolysosomes, thereby enhancing the proteasome-dependent pathway in the immune response. Herein, we first constructed the EV/ICG/MSN nanovaccine and then assessed the morphology, size, zeta potential, stability, and successively investigated nanovaccine uptake by DCs, the effects of laser-induced endolysosomal rupture, cytosolic delivery, DC maturation, and antigen presentation pathways. Finally, the *in vivo* protection effect was examined in animal experiments to investigate the biodistribution, immune response, prevention of drug-resistant *S. aureus*, and EV-coated multi-antigenic nanovaccine therapy.

## Experimental section

### Materials

Aminated magnetic MSN was obtained from So-Fe Biomedical Co., Ltd. (Shanghai, China). ICG was purchased from Meryer Chemical Technology Co., Ltd. (Shanghai, China). DiO, DiI, LysoTracker™ Red, and the BCA protein assay kit were purchased from the Beyotime Institute of Biotechnology (Shanghai, China). Primers used for the reactions were purchased from Wuhan Gene Create Biological Engineering Co., Ltd. (Wuhan, China). GM-CSF, IL-4 and 5(6)-CFDA N-succinimidyl ester (CFSE) were purchased from Abcam (Cambridge, UK). FITC-conjugated anti-mouse CD11c antibody was purchased from BioLegend (San Diego, USA). PE-conjugated CD86 monoclonal antibody, PE-conjugated CD80 monoclonal antibody, PE-conjugated CD40 monoclonal antibody, APC-conjugated CCR monoclonal antibody, APC-conjugated MHC-I monoclonal antibody, APC-conjugated MHC-II monoclonal antibody, PE-conjugated CD4 monoclonal antibody, APC-conjugated CD8 monoclonal antibody, and carboxy-H_2_DCFDA were purchased from Invitrogen (Carlsbad, USA). FITC-conjugated CD3 monoclonal antibody was purchased from eBioscience (CA, USA). Drug-resistant *S. aureus* BW15 and BWMR26 and drug-sensitive *S. aureus* S29213 strains were obtained from Dr. Gao (School of Medicine, Yangzhou University). Unless otherwise stated, all other reagents were purchased from the Nanjing Well Offer Biotechnology Co., Ltd. (Nanjing, China).

### Preparation and characterization of EVs from *S. aureus*

Drug-resistant *S. aureus* BW15 and BWMR26 28 and drug-sensitive *S. aureus* S29213 were cultured on Luria broth (LB) agar overnight at 37 °C and then a single colony was inoculated into LB medium on a rotary shaker. Then, a 1:100 dilution of the bacteria were cultured at 37 °C in LB medium until they reached late-logarithmic-phase. The bacterial culture was centrifuged at 6000 × g for 20 min to remove the bacteria, followed by filtering the medium through a 0.45 μm vacuum filter. The medium was then centrifuged at 150,000 × g for 2 h at 4 °C (Beckman Coulter, California, USA). The precipitates were considered the EV pellets 24. EV13, EV15, and EV26 were from *S. aureus* S29213, *S. aureus* BW15 and *S. aureus* BWMR26, respectively.

The EV particle morphology was monitored by transmission electron microscopy (TEM; Tecnai 12, Philips, Holland). The hydrodynamic size and potential were measured by Zeta Plus (Malvern Instruments, Worcestershire, UK). The production of EVs from each *S. aureus* strain was determined using a BCA protein assay and was shown as the total protein (mg) in EVs derived from 1 L of late-logarithmic-phase cultures. The total proteins in the EVs derived from different *S. aureus* strains were analyzed by SDS-PAGE and the protein abundance of the EV was analyzed by ImageJ 29.

### Preparation and characterization of EV-coated multi-antigenic nanovaccines

To improve the ICG loading efficacy, an aminated magnetic MSN was selected, which facilitated the binding of negatively charged ICG to MSN through electrostatic interactions. Briefly, 20 μg/mL of an ICG stock solution was prepared in water. Subsequently, MSN was dispersed in water and equal volumes of the ICG and MSN solutions were mixed and kept shaking for 2 h at room temperature in darkness. Finally, ICG/MSN was obtained by magnetic stand separation (Scheme 1A). The residual solution was measured by UV-Vis to estimate the ICG-loading capacity of the MSNs 30.

To generate the EV-coated nanoparticles, the ICG/MSN solution was mixed with excess EVs and then extruded through a 200 nm polycarbonate porous membrane with an Avanti mini-extruder. Following the extrusion, the EV-coated multi-antigenic particles (EV/ICG/MSN) were isolated from the excess EVs and soluble compounds by magnetic stand separation to obtain EV13/ICG/MSN, EV15/ICG/MSN, and EV26/ICG/MSN 31. The particle morphologies of ICG/MSN and EV/ICG/MSN were assessed by TEM. The hydrodynamic sizes and zeta potentials were measured by Zeta Plus. The protein concentrations of the nanoparticles before and after EV coating were quantified by BCA assay. The protein loading yield was defined as the weight ratio of immobilized proteins to the nanoparticles 32. The absorption spectra were recorded using a UV-Vis spectrophotometer, and the fluorescence spectra was recorded by fluorescence spectroscopy at an excitation wavelength of 700 nm. To test the photothermal effects of the nanovaccines *in vitro*, 100 μL of PBS, free ICG dissolved in an aqueous solution, the ICG/MSN solution, and three kinds of EV/ICG/MSN solutions containing 15 μg/mL of ICG were exposed to laser irradiation (808 nm, 0.8 W/cm^2^) for 5 min and the temperature change was recorded at 10 s intervals using a thermal probe. To evaluate the initial stability, EV13, EV15, EV26, MSN, ICG/MSN, and the three kinds of EV/ICG/MSN particles were incubated in PBS (pH 7.4) for 24 h and the changes in particle size were monitored by Zeta Plus.

### Cytotoxicity

Cytotoxicity was measured by CCK-8 assay in DC2.4 cells and BMDCs. BMDCs were isolated from the femurs and tibias of mice as previously described and cultured in RPMI 1640 complete medium supplemented with GM-CSF (20 ng/mL) and IL-4 (10 ng/mL) 33. A total of 1×10^5^ cells per well were seeded into 96-well plates, cultured for 12-16 h, and incubated with EVs, MSNs, and EV/ICG/MSN particles with increasing concentrations for 24 h. Subsequently, cells were incubated with CCK-8 solution and the UV-Vis absorbance was measured. Cell viability was normalized to PBS-treated cells and expressed as the means ± SD (n = 3). For the phototoxicity assay, cells were incubated with EV/ICG/MSN particles (20 μg/mL of total protein) for 4 h and washed 3 times with PBS, followed by irradiation with an 808 nm laser (0.8 W/cm^2^) for 30-180 s. Cells were cultured for another 24 h and cell viability was measured by CCK-8 assay.

### Cellular uptake, intracellular trafficking, and ROS detection

EVs were stained with DiI (λ_exc/em_ 549/565 nm) for cellular uptake by DC2.4 cells and BMDCs 34. EVs and EV/ICG/MSNs were added at a concentration of 20 μg/mL of total protein and incubated for 1, 2, and 4 h, respectively. Cells were then trypsinized and analyzed by flow cytometry to evaluate particle uptake. To evaluate endosomal escape, EVs were stained with DiO (λ_exc/em_ 484/501 nm). Cells were treated with EV/ICG/MSN and incubated for 2 h, followed by washing three times with PBS and then supplementation with fresh medium. Then, cells were then irradiated with the 808 nm laser (0.8 W/cm^2^) for 5 min and stained with LysoTracker™ Red for 30 min before imaging by confocal microscopy.

Intracellular ROS was detected using a H_2_DCFDA probe in BMDCs. Briefly, BMDCs were incubated with EV/ICG/MSN (20 μg/mL of total protein) at 37 °C for 4 h. Cells were washed three times with PBS and loaded with H_2_DCFDA diluted in fresh medium at 37 °C for 30 min. Then, cells were treated with or without 808 nm laser (0.8 W/cm^2^) irradiation for 3 min. ROS production was detected by flow cytometry.

For the proteasome activity assay, BMDCs were incubated with EV/ICG/MSN (20 μg/mL of total protein) with or without 3 μM of diphenylene iodonium (DPI, an NADPH oxidase inhibitor) pretreatment. Then, cells were treated with or without 808 nm laser (0.8 W/cm^2^) irradiation for 3 min. The proteasome activity was evaluated using a fluorometric assay kit (Biovision, CA, USA) according to the manufacturer's instruction.

### Maturation of DCs induced by EV-coated multi-antigenic nanovaccines

The isolated BMDCs were cultured in RPMI 1640 complete medium containing GM-CSF and IL-4. On day 6, immature BMDCs were seeded into a 12 well plate at a density of 10^6^ cells per well. Then, the cells were stimulated with 10 ng/mL of LPS, EV, or EV/ICG/MSN (20 μg/mL of total protein) for 48 h, with PBS used as a control. Subsequently, cells were washed and stained with fluorescent-labelled antibodies including FITC-CD11c, PE-CD86, PE-CD80, PE-CD40, APC-CCR, APC-MHC-I, APC-MHC-II, FITC-CD3, PE-CD4, and APC-CD8. Finally, all the samples were analyzed by flow cytometry.

After incubation with LPS, EV, and EV/ICG/MSN as described above, total RNAs were extracted from BMDCs using the trizol reagent. Then, the RNA was converted to cDNA by reverse transcriptase according to the manufacturer's instruction. The primer sequences were as follows: TNF-α, forward primer, 5'- GGA ACA CGT GGG ATA ATG-3', reverse primer, 5'- GGC AGA CTT TGG ATG CTT CTT-3'; IL-12, forward primer, 5'- AGA GGT GGA CTG GAC TCC CGA-3', reverse primer, 5'- TTT GGT GCT TCA CAC TTC AG-3'. Real-time qPCR analysis of mRNA expression was performed and the mRNA expression was normalized against GAPDH expression.

### Nanovaccine bio-distribution

To evaluate the bio-distribution of the nanoparticles *in vivo*, EVs were labelled with NHS-Cy5.5 (λ_exc/em_ 675/720 nm) 31. All animal experiments were conducted in accordance with the Animal Care and Use Committee of Yangzhou University. Pathogen-free 5-week-old male C57BL/6 mice were allowed to acclimate for one week before experiments (3 mice/group). Fluorescent-labelled EVs and EV/ICG/MSNs were injected subcutaneously at the tail-base site of mice as shown in Scheme 1A. The injection site was irradiated with a laser (808 nm, 0.8 W/cm^2^) for 5 min. The IVIS Lumina imaging system (Xenogen Co., USA) was used to detect the fluorescent signal *in vivo* at 0-24 h. The fluorescent imaging of inguinal LNs was performed at 24 h post-injection. IVIS live imaging software was used to analyze of the amount of fluorescent signal.

### *In vivo* immunization studies

5-week-old male C57BL/6 mice were randomly assigned to 4 groups for each of the EVs and allowed to acclimate for one week before experiments (6 mice per group). The mice were immunized subcutaneously at the tail base with 5 μg of EVs, EV/ICG/MSNs, and EV/ICG/MSNs with laser irradiation. The injection site was irradiated with a laser (808 nm, 0.8 W/cm^2^) for 5 min. Saline was used as control. The mice were immunized in week 0, 1, and 2 (Figure 6A). At the end of week 3, mice were euthanized and spleens were collected from each group. The isolated spleens were cut into pieces and mechanically digested into single cell suspensions. Then, the cells were lysed using red blood cell lysis buffer for 10 min. Subsequently, the splenocyte cells were resuspended and centrifuged at 1600 rpm at 4 °C for 5 min. Finally, 2×10^6^ splenocytes from each group were stained with fluorescent-labelled anti-mouse antibodies against CD3, CD4, and CD8. Then, all the samples were analyzed by flow cytometry. For the T lymphocyte proliferation assays, splenocytes were stained with 1 μM CFSE at 37 °C for 15 min, washed with RPMI 1640 medium, and seeded into 24-well plates (4 × 10^6^ cells per well) for 3 days of incubation. Subsequently, CD4^+^ and CD8^+^ T cells were stained and analyzed by flow cytometry. The CFSE-labeled T splenocytes cells were also restimulated *in vitro* with *S. aureus*, which were killed by formaldehyde fixation and thoroughly washed with PBS. After 2 days incubation, CD8^+^ T cells were stained and detected by flow cytometry. The potency of killing intracellular bacteria by restimulated CTL was determined performed according to the reported protocols 24,35. 2.5×10^4^ Raw 264.7 macrophage cells per well were seeded into 48-well plates, cultured for 12-16 h, and incubated with approximately 2.5×10^5^ colony forming units (CFU) of *S. aureus* for 1 h. Then the cells were washed with PBS and co-incubated with the restimulated splenocytes cells at the ratio 1:10 (macrophage: splenocytes). After 24 h co-incubation, the number of intracellular bacteria was determined by colony enumeration 35,36. For intracellular IFN-γ staining, cells were fixed and permeabilized after staining by CD8, and then stained with IFN-γ antibodies for further analysis as described in previous protocols 37. One day before the three immunizations and isolation of the spleen, serum was collected and used to quantified antibacterial IgG antibodies by an enzyme-linked immunosorbent assay (ELISA). EVs (200 ng/well) were used to coat 96-well plates, which were then blocked with 1% BSA. A total of 100 μL of diluted serum was loaded into the plate and incubated for 1 h at 37 °C. HRP-conjugated anti-mouse IgG antibodies (IgG total, IgG1 or IgG2a) were added to the wells and incubated for 1 h at 37 °C. Finally, the UV-Vis absorbance was measured at 450 nm.

### *S. aureus* infection and vaccine efficacy in prophylactic and therapeutic mouse models

For prophylactic experiments, 5-week-old male C57BL/6 mice were randomly assigned into the saline group, and EV/ICG/MSN + laser groups (6 mice per group) for each of the three types of EV. The immunization programmes were illustrated above (Figure 6A). The mice were immunized subcutaneously at the tail base with 5 μg of EV total protein/dose. The injection site was irradiated with a laser (808 nm, 0.8 W/cm^2^) for 5 min. After three immunizations, bacteria were subcutaneously inoculated into the abdomen, which was shaved before challenge. The immunized mice were challenged with 1 × 10^9^ CFU of bacteria on day 21. The lesion areas were recorded every day and calculated as the length multiplied by the width. On day 7 post-challenge the mice were euthanized, and the skin, heart, liver, spleen, lung, and kidney were isolated. The organs were subsequently homogenized in sterile PBS, diluted, and plated onto LB agar for colony enumeration after 24 h of incubation at 37 °C 38,39.

For therapeutic experiments, 5-week-old male C57BL/6 mice were randomly assigned to the saline group, antibiotic group, EV15/ICG/MSN + laser group, or EV15/ICG/MSN + laser + antibiotic group (6 mice per group). One day before immunization, bacteria were subcutaneously inoculated into the abdomen, which was shaved before challenge. The mice were challenged with 1 × 10^6^ CFU of *S. aureus* BW15. Then, the mice were immunized subcutaneously at the tail base with 5 μg of EV15 total protein/dose on days 0 and 7, respectively. The injection site was irradiated with a laser (808 nm, 0.8 W/cm^2^) for 5 min. As a control, 3 mg/kg of antibiotics were administered by intramuscular injection once a day on days 0, 1, and 2 (a total of 3 times). The lesion areas were recorded every day and calculated as the length multiplied by the width. On day 14 the mice were euthanized, and the skin, heart, liver, spleen, lung, and kidney were isolated for colony enumeration as illustrated above.

### Safety evaluation

The mice were administrated subcutaneously at the tail base with EV/ICG/MSNs under laser irradiation in week 0, 1, and 2 as illustrated in the in vivo immunization studies. Body weight of the mice was recorded every week. At the end of week 3, the main organs (heart, liver, spleen, lung, and kidney) and blood were collected from each group. The tissues were embedded in paraffin and 5 µm of sections were cut for H&E staining. Bloods were analyzed by Wuhan Servicebio Technology (Wuhan, China).

### Statistical analysis

Statistics were performed using GraphPad Prism 6 software. For the comparison of two groups a student's *t* test was used. For more than two groups a one-way ANOVA was used. ^*^P < 0.05, ^**^P < 0.01, ^***^P < 0.001, vs. control, saline or the relevant group, as illustrated in the Figure legends. Bacterial enumeration is shown as geometric mean ± SD. The other results are shown as the means ± SD.

## Results and discussions

### Preparation and characterization of EVs from *S. aureus*

**Figure 1:**
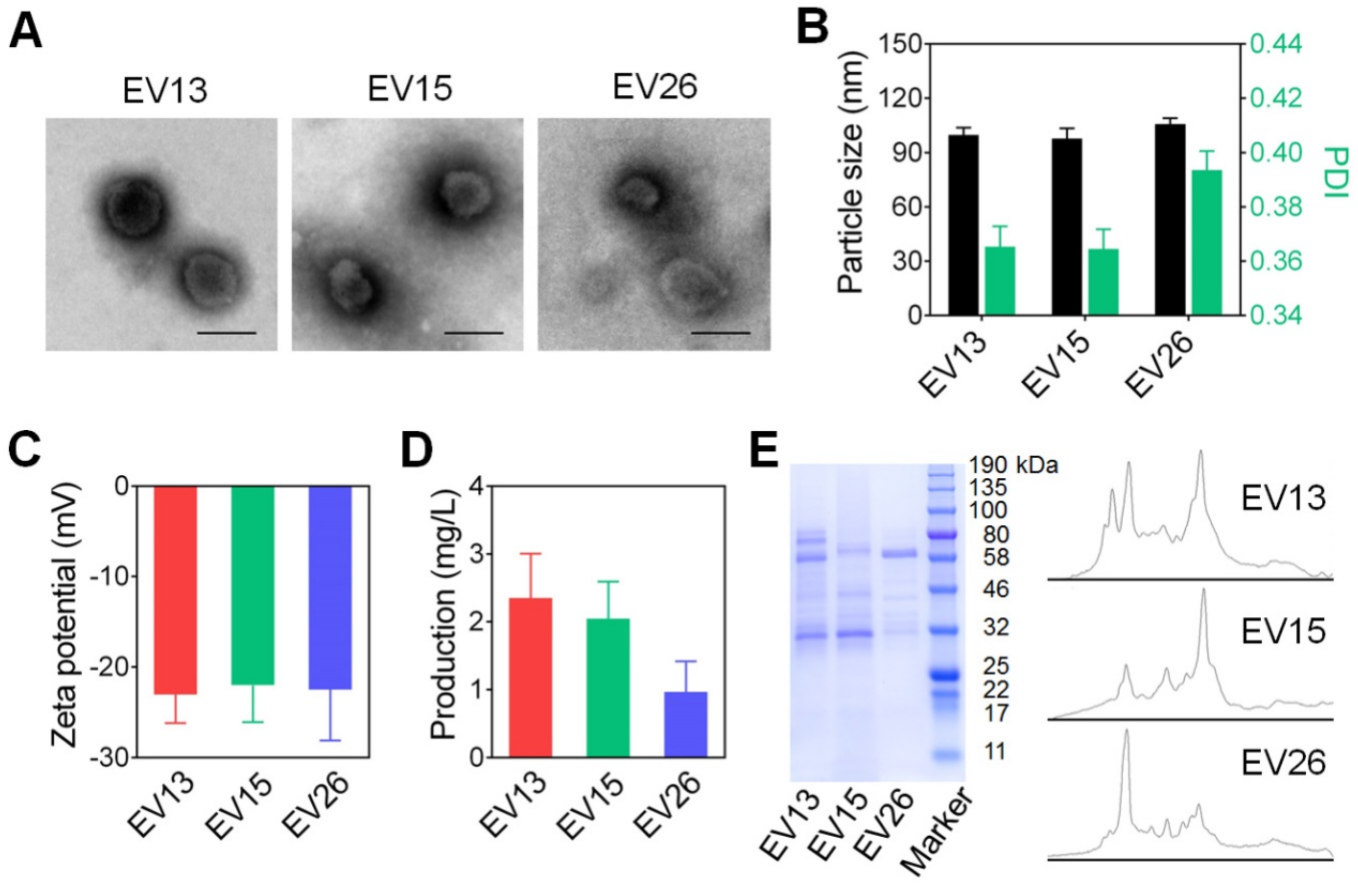
** Preparation and characterization of EVs from *S. aureus*. (A)** Representative TEM images of EV13, EV15 and EV26 from *S. aureus* S29213 (drug-sensitive), *S. aureus* BW15 (drug-resistant) and *S. aureus* BWMR26 (drug-resistant), respectively. Scale bar: 100 nm. **(B)** Hydrodynamic size (diameter, nm) and PDI of EVs. **(C)** Zeta potential of EVs. **(D)** The production of EVs from each *S. aureus* strain is shown as the total protein (mg) in EVs derived from 1 L of overnight culture, as determined by BCA protein assays. **(E)** SDS-PAGE analysis of EVs derived from different *S. aureus* strains. The molecular weights of a protein marker are indicated on the right. The protein abundance in the EVs was analyzed by ImageJ. Data are presented as the means ± SD (n = 6).

We first prepared EVs from drug-resistant *S. aureus* strains BW15 and BWMR26, and drug-sensitive *S. aureus* S29213 28. The obtained vesicles were named EV15, EV26, and EV13, respectively. The morphologies of representative EV15, EV26, and EV13 vesicles were evaluated by TEM (Figure 1A). All vesicles presented as spherical nanometer-sized particles with diameters of less than 100 nm. Particle size and zeta potential were measured by Zeta Plus as shown in Figure 1B-C. The diameters of EV13, EV15, and EV26 were 98.07 ± 0.21 nm, 95.77 ± 1.45 nm, and 108.65 ± 0.96 nm with polydispersity indexes (PDI) of 0.34 ± 0.01, 0.35 ± 0.01, and 0.39 ± 0.01, respectively (Figure 1B). These results were consistent with the sizes observed by TEM. All the vesicles displayed a negative surface charge (ζ potential) of approximately -20 mV (Figure 1C). The amount of EVs produced by the *S. aureus* strains was calculated as the total protein (mg) in EVs derived from 1 L of late-logarithmic-phase cultures and analyzed by a BCA protein assay. As shown in Figure 1D, the amount of EV13 and EV15 was similar, which was approximately two-fold higher than that of EV26. In addition, the protein components incorporated into each type of EV was strain-dependent (Figure 1E).

### Preparation and characterization of EV-coated multi-antigenic nanovaccines

**Figure 2:**
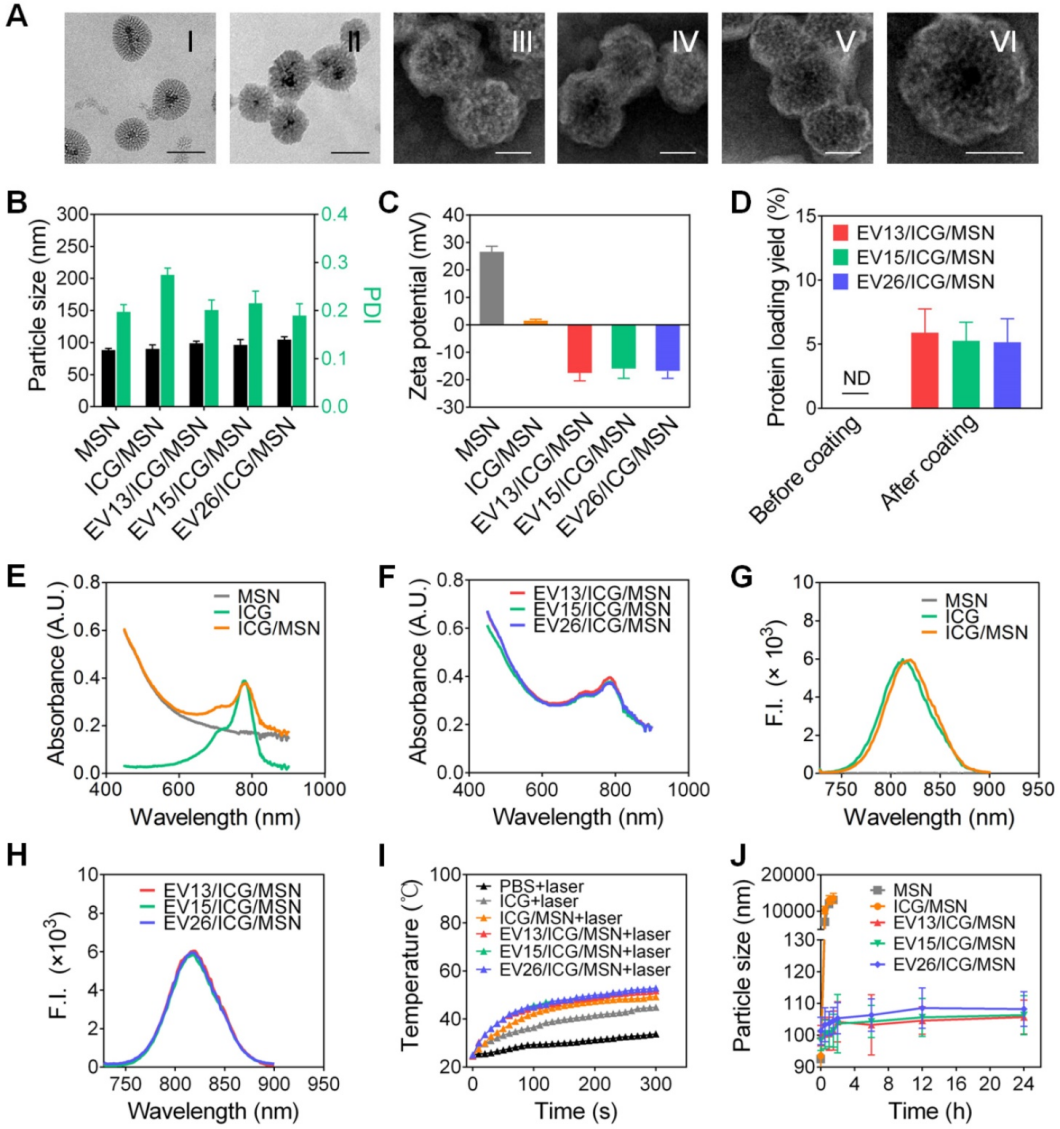
** Preparation and characterization of EV-coated multi-antigenic nanovaccines. (A)** Representative TEM images of MSN (I, scale bar: 100 nm), ICG/MSN (II, scale bar 100 nm), EV13/ICG/MSN (III, scale bar: 50 nm), EV15/ICG/MSN (IV, scale bar: 50 nm), EV26/ICG/MSN (V, scale bar: 50 nm) and a zoomed-in view of a single EV/ICG/MSN nanoparticle (VI, scale bar: 50 nm). **(B)** Hydrodynamic size (diameter, nm), PDI and **(C)** zeta potential of MSN, ICG/MSN, EV13/ICG/MSN, EV15/ICG/MSN and EV26/ICG/MSN. **(D)** Quantification of protein concentrations of nanovaccines before and after membrane coating. **(E)** Absorption spectra of MSN, ICG, ICG/MSN. **(F)** Absorption spectra of EV-coated hybrid nanovaccines. **(G)** Fluorescence spectra of MSN, ICG, ICG/MSN. **(H)** Fluorescence spectra of EV-coated hybrid nanovaccines. **(I)** Solution temperature changes after exposure to laser irradiation. **(J)** Size changes of MSN, ICG/MSN and EV-coated nanovaccines in PBS buffer. Data are presented as the means ± SD (n = 3).

Aminated magnetic MSN was chosen for the preparation of EV-coated multi-antigenic nanovaccines. The positive surface charge of MSN facilitates the loading of negatively charged ICG. As shown in Figure 2A (I), mesoporous structures could be observed on the MSNs and the pore width was 4.89 nm, as determined by nitrogen adsorption assays (Figure S1). After ICG loading, the mesoporous structures became ambiguous (Figure 2A (II)). We then used EVs from each S. aureus strain to coat the ICG-loaded MSNs. All the coated particles displayed spherical structures enclosed in a thin shell (Figure 2A (III)-(VI)). The EV/ICG/MSNs had a slightly larger hydrodynamic size than the MSNs and ICG/MSNs with the addition of a lipid membrane less than 10 nm thick (Figure 2B). Meanwhile, the zeta potentials of the MSNs and ICG/MSNs were 26.62 ± 2.01 mV and 1.51 ± 0.58 mV, respectively. The decreased surface charge observed after ICG loading should be due to the two sulfonic groups in each ICG molecule 30. However, after coating by EVs, the zeta potentials of EV13/ICG/MSN, EV15/ICG/MSN, and EV26/ICG/MSN were -17.52 ± 2.87 mV, -15.93 ± 3.54 mV, and -16.81 ± 2.64 mV, respectively (Figure 2C). The surface charge of the EV/ICG/MSNs was slightly more positive than the EVs (Figure 1C). The membrane coating was further verified by a BCA assay (Figure 2D). The ICG/MSNs had a negligible adsorption of protein before being coated with vesicles. By contrast, the three EV/ICG/MSNs displayed a significant increase in their UV-Vis absorbance at 562 nm implying the presence of protein adsorption. The quantification of protein loading yield showed an increase of 5.89%, 5.27%, and 5.16% for EV13/ICG/MSN, EV15/ICG/MSN, and EV26/ICG/MSN, respectively. Protein electrophoresis indicated that the membrane proteins from the EVs could be well retained on the EV/ICG/MSNs (Figure S2A). The UV-Vis absorbance spectra and fluorescence spectra of free ICG, MSN, ICG/MSN, and EV/ICG/MSN showed that the absorption and emission peaks were located at 780 nm and 810 nm, respectively, which was consistent with the absorption/emission peak of ICG (Figure 2E-H). Meanwhile, only MSN possessed no absorption or emission peaks. These results confirmed that ICG was successfully loaded and EVs were coated onto the exterior surface of MSNs.

Next we evaluated the photothermal effects of EV/ICG/MSN in vitro. Changes in the solution temperature were recorded for 5 min following irradiation with an 808 nm laser (0.8 W/cm^2^, Figure 2I). The results showed superior photothermal effects for all of the EV/ICG/MSN particles, as documented by the ability to reach more than 50 °C during the irradiation period. Conversely, in terms of photothermal effects, free ICG had a significantly lower temperature increase compared to the same mass of ICGs encapsulated in ICG/MSNs. As a control, phosphate-buffered saline (PBS) showed negligible heating under the same experimental conditions. The reason why EV/ICG/MSNs have good photothermal efficiency is that the ICGs encapsulated in EV-coated MSNs with a high packing density can absorb light and release of heat with extremely high efficiency 40.

To determine whether ICG loading and EV coating affect the colloidal stability of MSNs, we used PBS to simulate physiologic ionic conditions to study the colloidal stability of the particles. Changes in the hydrodynamic size of MSNs, EVs, ICG/MSNs, and EV/ICG/MSNs were monitored for 24 h in PBS (Figures 2J, S2B). The results showed that MSNs and ICG/MSNs tended to aggregate, as documented by the increase in size to larger than 10 µm at 1.5 h (Figure 2J). The EVs without inner cores also showed an increase in size from 100 nm to 300 nm within 24 h in PBS (Figure S2B). In contrast, the size changes for all three kinds of EV-coated nanoparticles were negligible, confirming that membrane coating effectively stabilized the nanoparticles against aggregation 25.

### Uptake and enhanced cytosolic delivery of EV-coated multi-antigenic nanovaccines by DCs

Antigen uptake by antigen presenting cells (APCs; DCs are the most potent and crucial APCs) is the first step in the generation of potent immune responses 35. However, the cytotoxicity of EV-coated multi-antigenic nanovaccines needs to be taken into account before evaluating their uptake by DCs. First, we evaluated the cytotoxicity of EVs in bone marrow-derived DCs (BMDCs). As expected, none of the three types of EVs induced any decrease in cell viability even at high protein concentrations of 50 μg/mL (Figure S3A). We then evaluated the cytotoxicity of MSNs in BMDCs. As shown in Figure S3B, the cell viability remained above 95% at concentrations of 500 μg/mL of MSN. When the concentration was increased to 1000 μg/mL, MSNs led to 30% cell death. Subsequently, we evaluated the cytotoxicity of EV/ICG/MSNs in BMDCs and DC2.4 cells (Figure S3C-D). There was no obvious cytotoxicity induced by any of the three types of nanovaccines, suggesting that the EV-coated multi-antigenic nanovaccines had no adverse effects, at least in the settings of this experiment. Furthermore, the phototoxicity of EV/ICG/MSNs was measured by exposing them to laser irradiation (808 nm, 0.8 W/cm^2^) for different periods of time (Figure S3E). None of the EV/ICG/MSNs exhibited any substantial cytotoxicity after 180 s at total protein concentrations of 20 μg/mL. These cellular cytotoxicity results were used as guidance for subsequent cell experiments.

**Figure 3:**
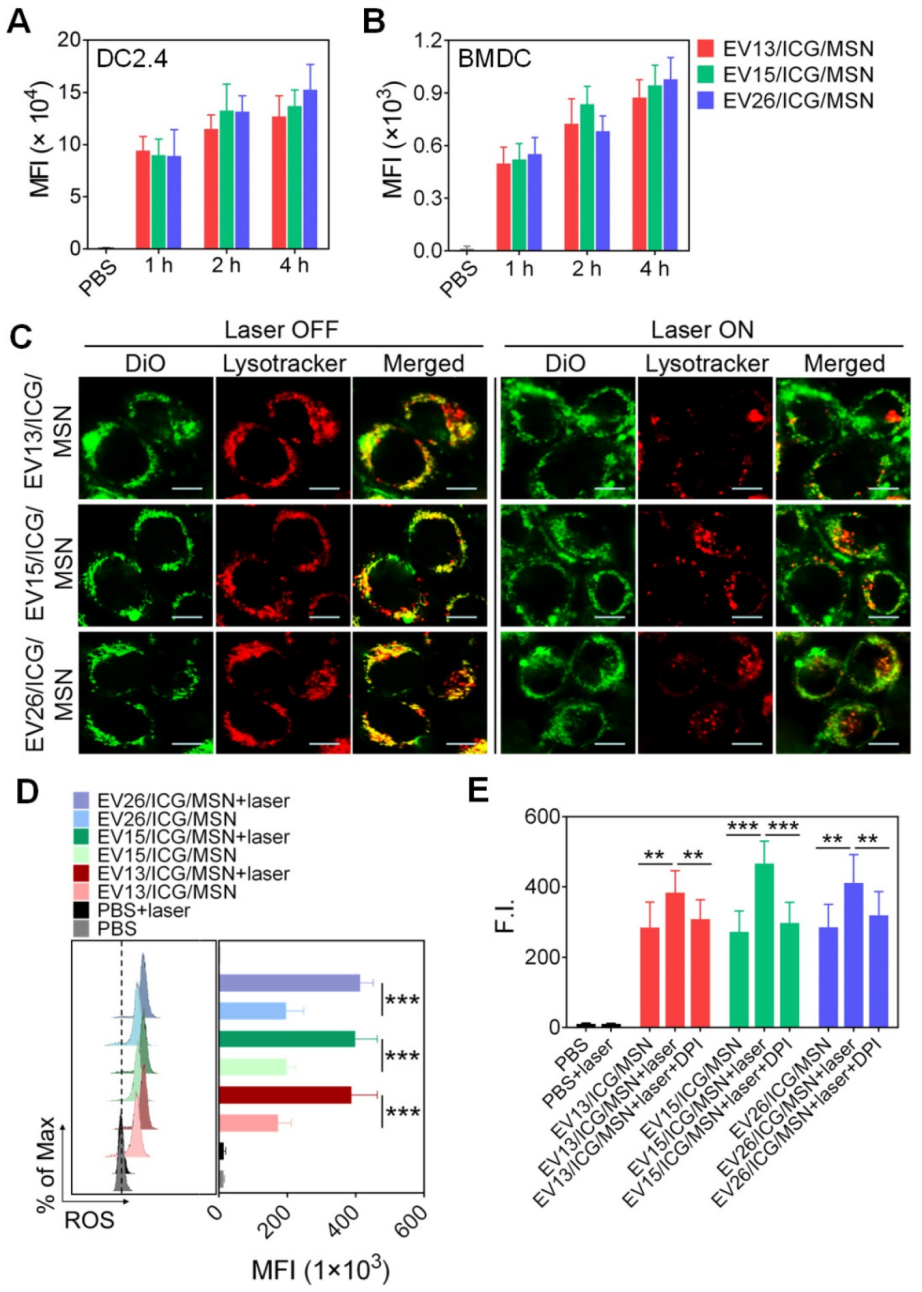
** Intracellular trafficking of EV-coated multi-antigenic nanovaccines and ROS generation for enhancing proteasome activity. (A-B)** Cell uptake by DC2.4 cells and BMDCs determined by flow cytometry at 1, 2 and 4 h post-incubation with EV-coated nanovaccines. **(C)** Photothermally triggered endosomal escape. Confocal images of BMDCs treated with EV-coated nanovaccines without (left) and with (right) laser irradiation. Endosomes were stained with LysoTracker™ Red. EVs were labelled with DiO (green). Scale bar: 10 µm. **(D)** Effect of EV-coated hybrid nanovaccines on ROS generation in BMDCs under laser irradiation using H_2_DCFDA as an ROS tracker that was quantified by flow cytometry. **(E)** Proteasome activity in BMDCs was analyzed by a fluorometric assay kit. Data are presented as the means ± SD (n = 3). ^**^P < 0.01, ^***^P < 0.005, vs the indicated groups.

Cellular uptake of EV/ICG/MSNs was performed in DC2.4 cells and BMDCs. As expected, cellular uptake increased following longer incubation times at total protein concentrations of 20 μg/mL (Figure 3A-B). No significant differences were observed in uptake among EV13/ICG/MSN, EV15/ICG/MSN, and EV26/ICG/MSN. However, EV/ICG/MSNs showed higher efficacy in delivering EVs into DCs (Figure S4), which may have benefited from the superior stability in particle size and the rigidity of EV-coated particles (Figures 2J, S2B). Smaller-sized nanoparticles are taken up more efficiently by DCs. Moreover, rigid particles are more easily wrapped by the cell membrane, whereas flexible particles are deformed by the membrane, resulting in increased energy expenditure and consequently decreased cellular uptake 41. We next assessed intracellular trafficking by colocalization analysis of DiO-labelled EV/ICG/MSNs with LysoTracker™ Red. After endocytosis of EV/ICG/MSNs, heat was expected to be generated from ICG by laser irradiation, which would lead to the disruption of the endosomal membranes and the release of the particles into the cytoplasm. To validate this hypothesis, we evaluated differences in intracellular trafficking with or without laser irradiation. As shown in Figure 3C, in the absence of laser irradiation, most of the endocytosed particles were located in lysosomes, as indicated by the overlapping yellow fluorescence between DiO and LysoTracker. However, most of the DiO fluorescence separated from the LysoTracker signal after laser irradiation, suggesting efficient endosomal escape and cytosolic release. This indicates that ICGs encapsulated in MSNs could induce the MHC-I pathway of antigen presentation after laser irradiation 42.

Recent studies showed that lysosome disruption could directly trigger ROS production in DCs, which was found to be essential for enhancing proteasome activity and downstream MHC-I antigen presentation 43. We next evaluated ROS production and proteasome activity in BMDCs upon the disruption of the endosomal membrane triggered by the heat of EV-coated nanovaccines. As shown in Figure 3D, all three classes of EV/ICG/MSN showed much higher levels of ROS after laser irradiation compared to untreated EV/ICG/MSNs (*P* < 0.005). Further assays showed that the proteasome activity was significantly improved when EV/ICG/MSNs were combined with laser irradiation (Figure 3E,* P* < 0.01 or *P* < 0.005). When ROS generation was inhibited by DPI, the proteasome activity was remarkably attenuated even with laser irradiation. These data suggest that ROS generation from lysosome disruption triggered by EV/ICG/MSN upon laser irradiation contributed to the enhanced proteasome activity. The efficient endosomal escape and improved proteasome activity were both involved in antigen presentation by the proteasome-dependent pathway.

### EV-coated multi-antigenic nanovaccines promote DC maturation and modulate antigen presentation pathways

**Figure 4:**
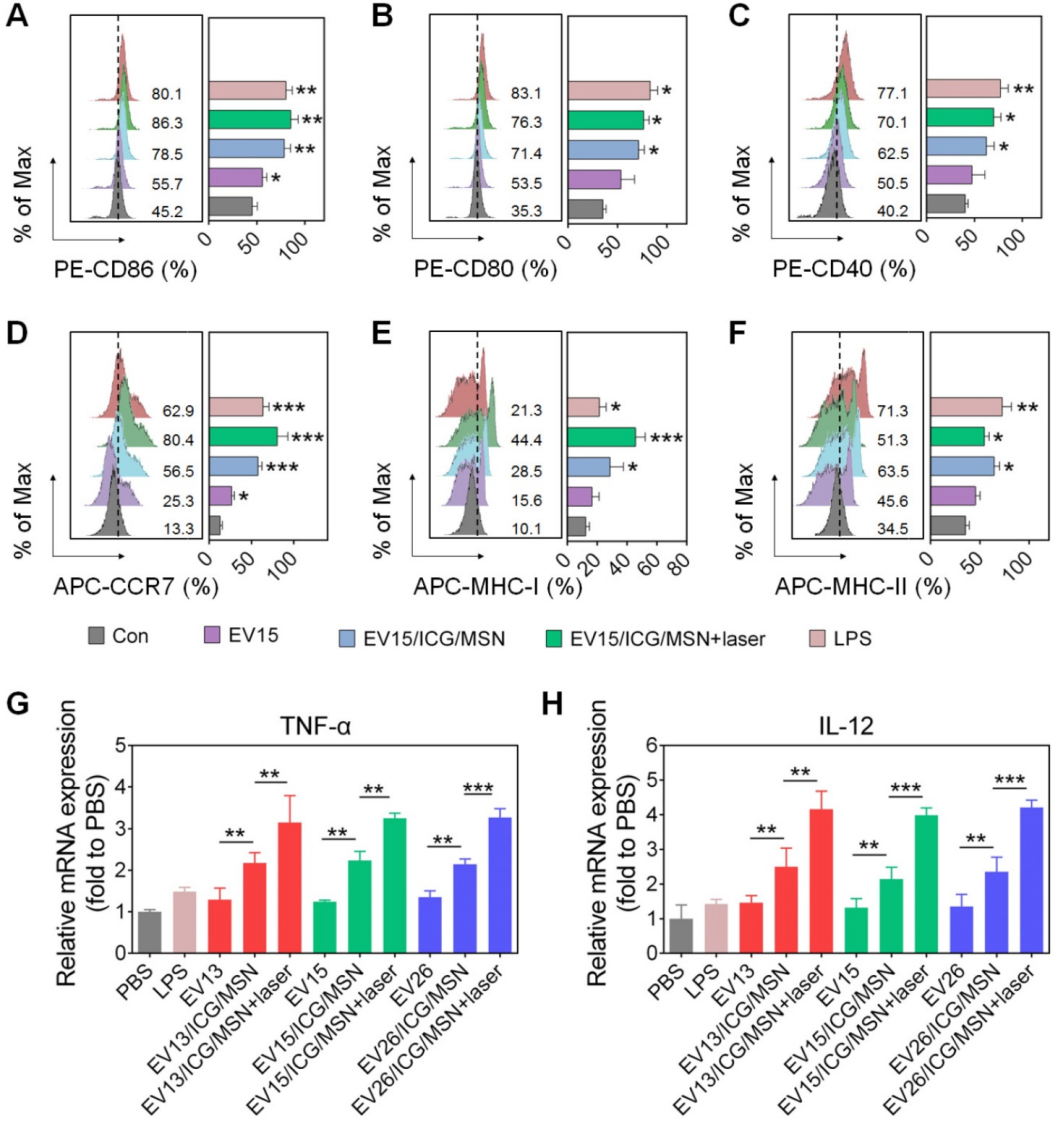
** DC cellular maturation induced by EV-coated multi-antigenic nanovaccines. (A-F)** Percentage of positive cells of CD86, CD80, CD40, CCR7, MHC-I and MHC-II expression by BMDCs was determined by flow cytometry. **(G-H)** Quantitative RT-PCR analysis of TNF-α and IL-12 in BMDCs. Data are presented as the means ± SD (n = 3). *P < 0.05, **P < 0.01, ***P < 0.005, vs the control or indicated groups.

The activation of dcs into mature cells is indispensable for the initiation of immune responses. Mature dcs upregulate expression levels of characteristic markers, such as the costimulatory molecules, MHC-I, MHC-II and cytokines 44. As shown in Figure 4A-C and Figure S5, EV15/ICG/MSN with or without laser irradiation greatly elevated the expression levels of CD86 (without laser, 78.5%; with laser, 86.3%), CD80 (without laser, 71.4%; with laser, 76.3%), and CD40 (without laser, 62.5%; with laser, 70.1%) compared to EV15 alone (CD86, 55.7%; CD80, 53.5%; CD40, 50.5%), which was comparable to LPS treatment (CD86, 80.1%; CD80, 83.1%; CD40, 77.1%). These phenomena can be attributed to the superior stability and rigidity of EV-coated particles (Figures 2J, S2B) and the subsequent improvement in cellular uptake (Figure S4). Under laser irradiation, EV15/ICG/MSN did not significantly increase in the expression of CD86, CD80, or CD40. Additionally, treatment of EV15/ICG/MSN with laser irradiation upregulated the expression of CCR7 (80.4%) by 56.5% for EV15/ICG/MSN, compared to a 25.3% increase for EV15 and a 62.9% increase for LPS (Figure 4D). These results suggested that EV15/ICG/MSN with laser irradiation enhanced the migration capacity of dcs, which is beneficial for their accumulation in the lymph nodes (lns).

The presentation of antigens through MHC class I, known as the proteasome-dependent pathway, is essential for the initiation of CD8^+^ T cell responses. By contrast, MHC-II presents antigens to CD4^+^ T cell via the lysosome-dependent pathway 45. In the present study, BMDCs expressed significantly higher levels of MHC-I on their surfaces after incubation with the EV15/ICG/MSN upon laser irradiation (44.4%) compared to EV treatment (15.6%) or EV15/ICG/MSN in the absence of laser irradiation (28.5%), even compared to LPS (21.3%) (Figure 4E). For MHC-II expression, EV15/ICG/MSN resulted in slightly less MHC-II expression under laser irradiation compared to that of untreated EV15/ICG/MSN. However, compared to EVs, the expression of MHC-II was higher after treatment with EV15/ICG/MSN with or without laser irradiation (Figure 4F). Similar results were observed in other two kind EVs (Figure S6 and S7). Hence, the proteasome-dependent pathway was enhanced by endosomal escape, improved proteasome activity, and elevated MHC-I expression, while MHC-II antigen presentation was not significantly compromised in the lysosome-dependent pathway.

The Th1-polarizing cytokines TNF-α and IL-12 are extremely important for eliciting protective cellular immune responses 27. We next determined whether activation also promoted cytokine generation during DC maturation. As shown in Figure 4G-H, the expression levels of the transcripts encoding TNF-α and IL-12 were significantly elevated after treatment of EV/ICG/MSNs with laser irradiation compared to those without laser irradiation (*P* < 0.01 or *P* <0.005), which was still higher than EVs (*P* < 0.01). This immune response could promote the activation of CD8^+^ T cells and CTL responses.

### *In vivo* tracking of EV-coated multi-antigenic nanovaccines

**Figure 5:**
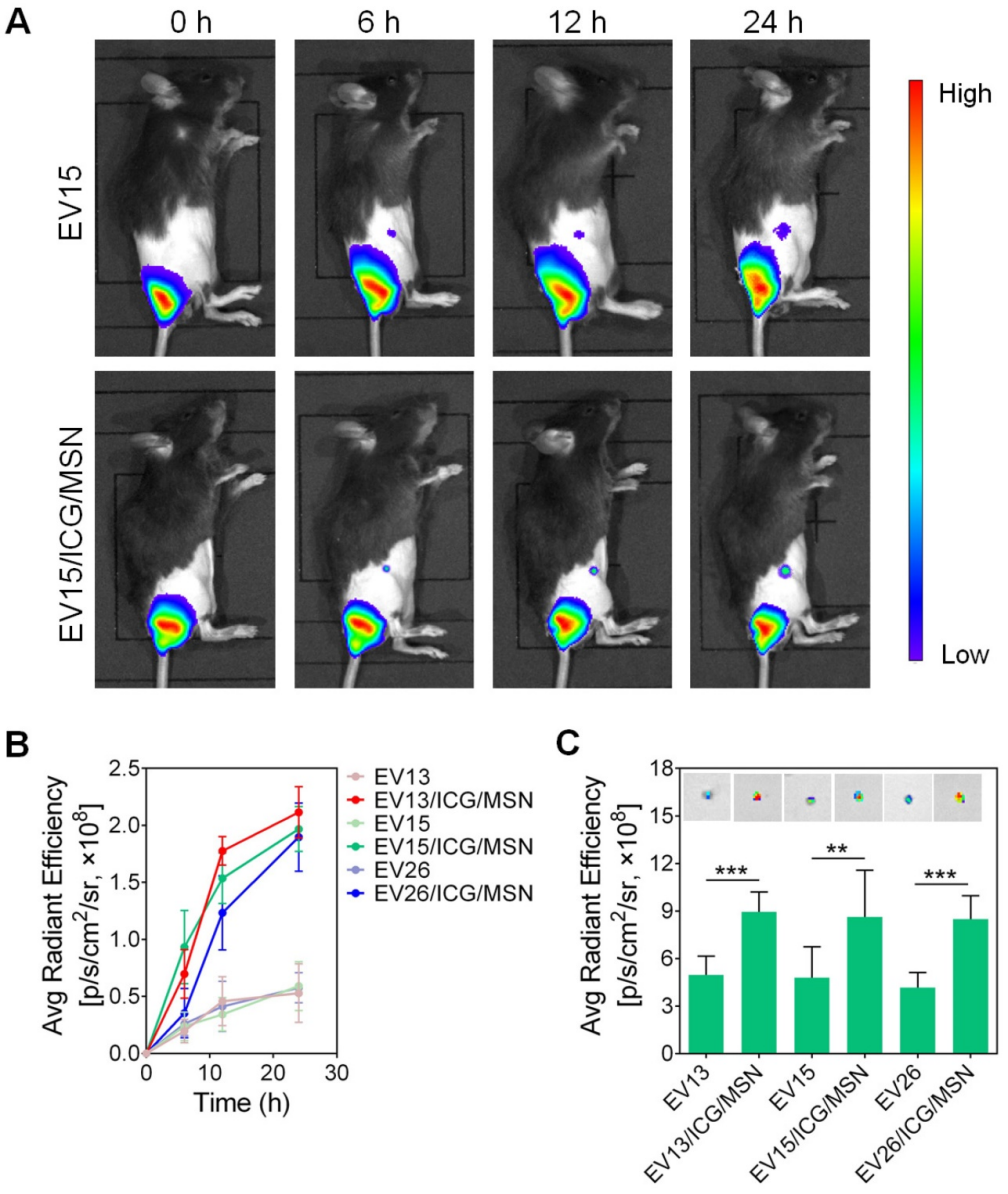
***In vivo* tracking of EV-coated multi-antigenic nanovaccines at designated time intervals post-injection. (A)** *In vivo* fluorescence images and **(B)** fluorescence intensity in draining lymph nodes (LNs) of EV and EV/ICG/MSN at different time points. **(C)** Representative *ex vivo* fluorescence images and fluorescence intensity of the draining LNs. Data are presented as the means ± SD (n = 3). **P < 0.01, ***P < 0.005, vs the indicated groups.

To investigate the transportation of EV-coated multi-antigenic nanovaccines, mice were vaccinated at their tail-base site and monitored by an *in vivo* imaging system. As shown in Figure 5A, S8, significantly increased inguinal LN accumulation was observed for EV/ICG/MSNs compared to EVs. Quantification of LN-associated fluorescence showed initial fluorescent signals in the inguinal LN area at 6 h post-injection, with continuous increases in LNs accumulation. Strong fluorescence could be observed in the LNs of animals treated with the EV/ICG/MSN (Figure 5B). *Ex vivo* evaluation confirmed that the fluorescence was approximately 2-fold higher in isolated LNs from EV/ICG/MSN-vaccinated mice compared to EV-vaccinated mice at 24 h (*P* < 0.01, *P* < 0.005) (Figure 5C). These results suggested that there was efficient transportation of EV-coated multi-antigenic nanovaccines from the injection site into LNs.

### EV-coated multi-antigenic nanovaccines stimulated potent immune response *in vivo*

**Figure 6:**
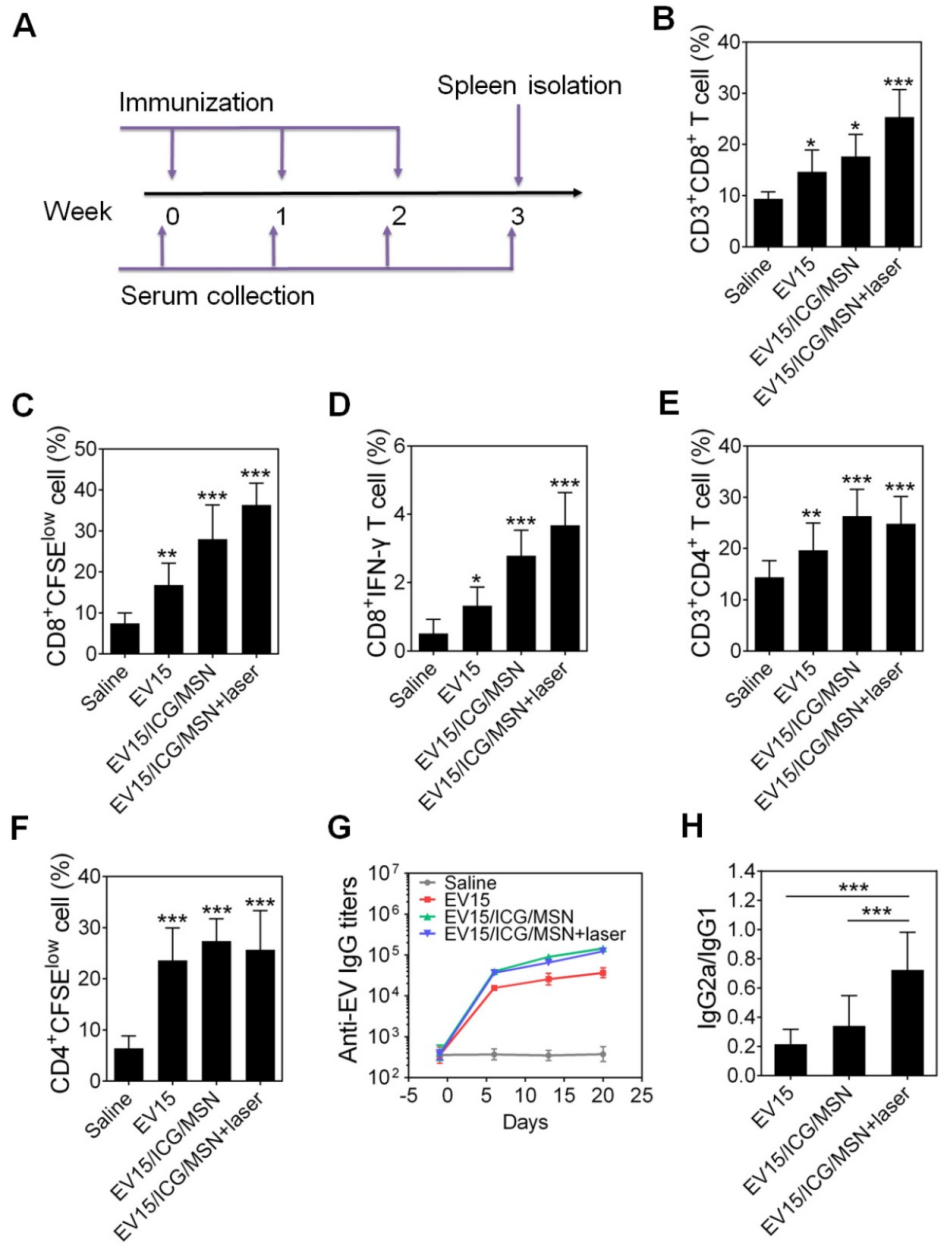
** EV-coated multi-antigenic nanovaccines elicited a potent immune response *in vivo*. (A)** Study protocol for immunizations and evaluations. **(B)** The proportion of CD3^+^CD8^+^ T cells in splenocytes was determined by flow cytometry. **(C)** The proliferation of CD8^+^ T cells was assessed by CFSE dilution. **(D)** The proportion of IFN-γ-producing CD8^+^ T cells was determined by flow cytometry. **(E)** The proportion of CD3^+^CD4^+^ T cells in splenocytes was determined by flow cytometry. **(F)** The proliferation of CD4^+^ T cells was assessed by CFSE dilution. **(G)** Time course of EV-specific IgG titers. **(H)** The ratio of IgG2a/IgG1 was measured on day 21 in sera from immunized mice. Data are presented as the means ± SD (n = 6). *P < 0.05, **P < 0.01, ***P < 0.005, vs the saline or indicated groups.

As a potential nanovaccine, the immune response of the EV-coated multi-antigenic nanovaccines was assessed *in vivo*. The protocol for the immunizations and evaluations is shown in Figure 6A. CD8^+^ T cells are an important component of protection against intracellular infections 27. In the present study, the percentage of CD3^+^CD8^+^ T lymphocytes was measured using flow cytometry. As shown in Figure 6B, S9A, and S10A, all three types of EV/ICG/MSNs significantly enhanced the CD8^+^ T cell proportion upon laser irradiation (EV15/ICG/MSN, 25.4%; EV13/ICG/MSN, 22.4%; EV26/ICG/MSN, 19.3%) compared to the saline group (9.4%), the EV group (EV15, 14.6%; EV13, 13.7%; EV26, 11.6%) and the EV/ICG/MSN group without laser irradiation (EV15/ICG/MSN, 17.6%; EV13/ICG/MSN, 15.0%; EV26/ICG/MSN, 13.4%). These results could be explained by the increased CD8^+^ T cellular proliferation upon immunization with EV/ICG/MSN under laser irradiation (Figure 6C, S9B, and S10B). Furthermore, to evaluate the activation of CD8^+^ T cells, we quantified the number of intracellular interferon-γ (IFN-γ)-producing CD8^+^ T cells. As shown in Figure 6D, S9C, and S10C, intracellular cytokine staining showed that immunization with EV/ICG/MSN resulted in high levels of CD8^+^IFN-γ^+^ T cells (EV15/ICG/MSN, 3.7%; EV13/ICG/MSN, 3.4%; EV26/ICG/MSN, 3.9%), which was higher than that elicited by EVs (EV15, 1.3%; EV13, 1.0%; EV26, 1.3%) or EV/ICG/MSNs without laser irradiation (EV15/ICG/MSN, 2.8%; EV13/ICG/MSN, 2.5%; EV26/ICG/MSN, 2.4%). Then, the effects of immunization on activation of bacterium-specific T cell responses were investigated. Splenocytes were stained with CFSE and restimulated by inactivated bacteria to monitor CTL proliferation. As shown in Figure S11A, fluorescent intensity of CD8^+^ T cells from EV15/ICG/MSN with laser irradiation group decreased significantly than the saline group. A ratio of 15:1 fluorescence intensity of EV15/ICG/MSN with laser irradiation versus saline group displayed CD8^+^ T cellular proliferation, faster than EV15/ICG/MSN and EV15 groups. Subsequently, killing of intracellular *S. aureus* by specific CTL was examined. As shown in Figure S11B, EV15/ICG/MSN with laser irradiation group could generate considerable cytotoxic effect against intracellular bacteria. Altogether, immunization with EV/ICG/MSN under laser irradiation was able to induce robust bacterium-specific CD8^+^ T cell responses, which was likely due to the superior stability and rigidity, improved cellular uptake and cytosolic delivery efficacies, elevated MHC-I expression, and improved proteasome activity of DCs.

CD4^+^ T cells play a critical role in modulating both cellular and humoral immunity 46,47. The proportion and proliferation of CD4^+^ T cells were determined by flow cytometry. As shown in Figure 6E, 6F, S9D, S9E, S10D, and S10E, the percentage of CD4^+^ T cells was slightly decreased with immunization by EV/ICG/MSN with laser irradiation (EV15/ICG/MSN, 24.8%; EV13/ICG/MSN, 23.1%; EV26/ICG/MSN, 23.3%) compared to those without laser irradiation (EV15/ICG/MSN, 26.3%; EV13/ICG/MSN, 25.3%; EV26/ICG/MSN, 25.3%), but was still higher than that induced by EV treatment (EV15, 19.6%; EV13, 17.3%; EV26, 17.0%). To investigate the effects of EV/ICG/MSNs on antibody responses, anti-EV IgG titers were measured. Comparable levels of antigen-specific IgG titers were induced by EV/ICG/MSNs with or without laser irradiation, which were both higher than those induced by EVs (Figure 6G, S9F, and S10F). These results demonstrate that immunization with EV/ICG/MSN under laser irradiation induced improved CD8^+^ T cell responses, while at the same time maintaining CD4^+^ T cells responses and humoral immunity.

Antibody isotypes determine the type of T helper (Th) cell immune responses. IgG2a and IgG1 are markers for Th1 and Th2 cells, respectively. The ratios of IgG2a/IgG1 of each group were analyzed (Figure 6H, S9G, and S10G). EV/ICG/MSN with laser irradiation induced significantly higher IgG2a/IgG1 ratios than EVs (*P* < 0.005) and EV/ICG/MSN (*P* < 0.005) without laser irradiation, which indicated that immunized mice were Th1 polarized.

### EV-coated multi-antigenic nanovaccines reduced *S. aureus* infection in prophylactic experiment

**Figure 7:**
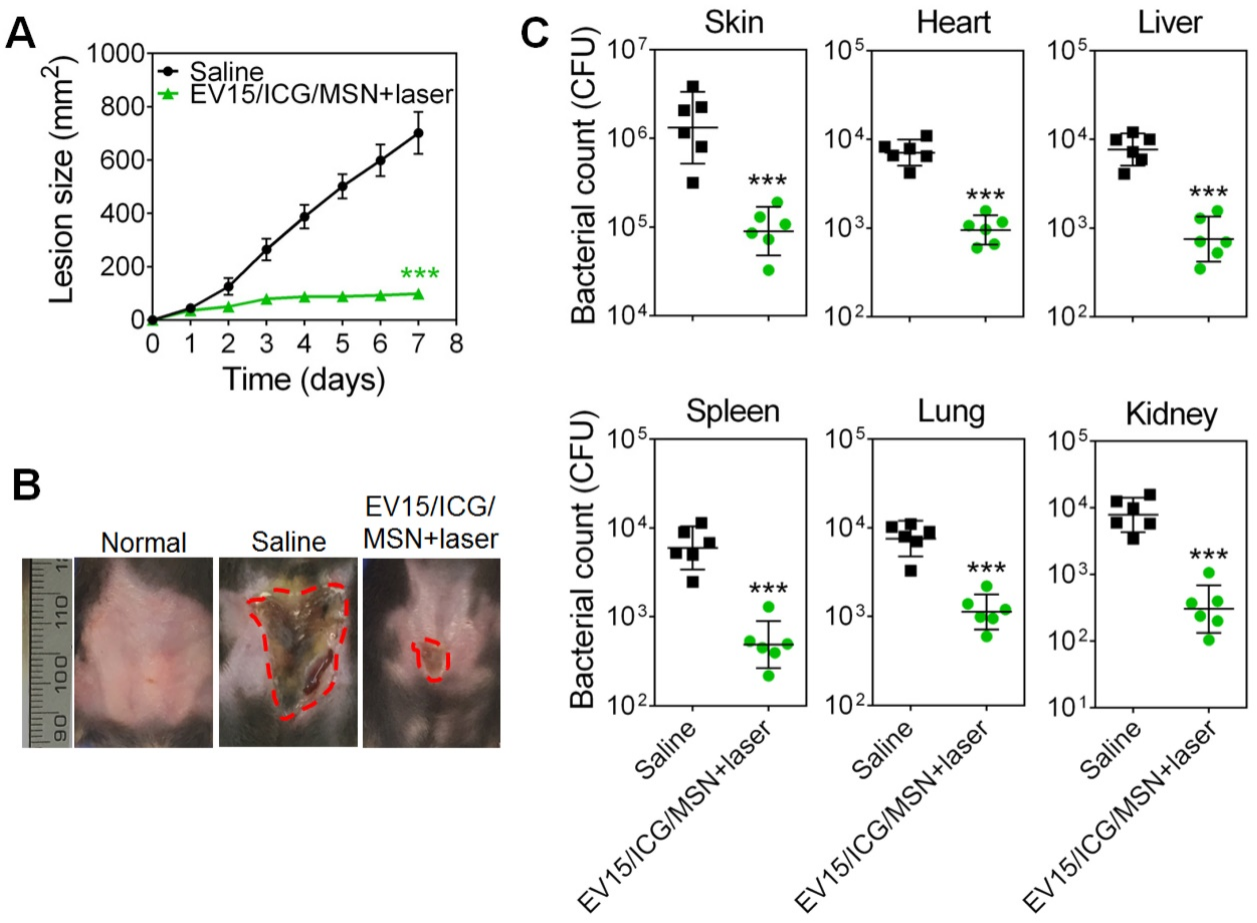
** EV-coated multi-antigenic nanovaccines-induced prophylactic immune effects in *S. aureus* BW15 infected mouse models. (A)** Skin lesion sizes were monitored over the course of infection (means ± SD; n = 6). **(B)** Images of skin lesions from different groups on day 7. **(C)** The infected skin and major organs, including the heart, liver, spleen, lung, and kidney were collected and the bacterial burdens were enumerated. Bacterial enumeration data are presented as the geometric mean ± SD (n = 6). ^*^P < 0.05, **P < 0.01, ***P < 0.005, vs the saline group.

We then evaluated the protective capability of EV-coated multi-antigenic nanovaccines in preventing bacterial infection by employing drug-resistant *S. aureus* BW15 and BWMR26 and sensitive *S. aureus* S29213 in the development of a skin infection model 28. Immunization was carried out with the EV-coated nanovaccines prepared from these three bacterial strains. The progression of skin lesion development in mice was monitored for 7 days. Immunization by EV/ICG/MSN with laser irradiation significantly inhibited lesion formation when compared with that of the saline group, indicating effective *S. aureus* prevention among the drug-resistant and drug-sensitive *S. aureus* strains (Figure 7A, S12A, and S13A). The images of skin lesions in Figure 7B confirm the lesion size measurements. Furthermore, the bacterial burden in the infected skin area and disseminated infection were further quantified (Figure 7C, S12B, and S13B). For the saline group challenged with *S. aureus* BW15, S29213, and BWMR26 strains, the bacterial burdens of the infected skin tissues were 1.7 × 10^6^, 2.0 × 10^6^, and 1.6× 10^6^ CFU, respectively. Mice immunized with EV/ICG/MSN under laser irradiation showed significant reductions compared with saline group (*P* < 0.005). As shown in Figure 7C, S12B, and S13B, disseminated infection was evaluated by quantification of bacterial counts in the heart, liver, spleen, lung, and kidney. Groups given EV/ICG/MSN with laser irradiation showed a significant drop in their bacterial burdens compared with that of the saline groups. Overall, these results demonstrate that EV-coated multi-antigenic nanovaccines under laser irradiation not only reduced superficial infection but also decreased bacterial invasiveness, which can be beneficial to avoid complications associated with *S. aureus* infections.

### EV-coated multi-antigenic nanovaccines achieved therapeutic effects with drug-resistant *S. aureus* infection models

**Figure 8:**
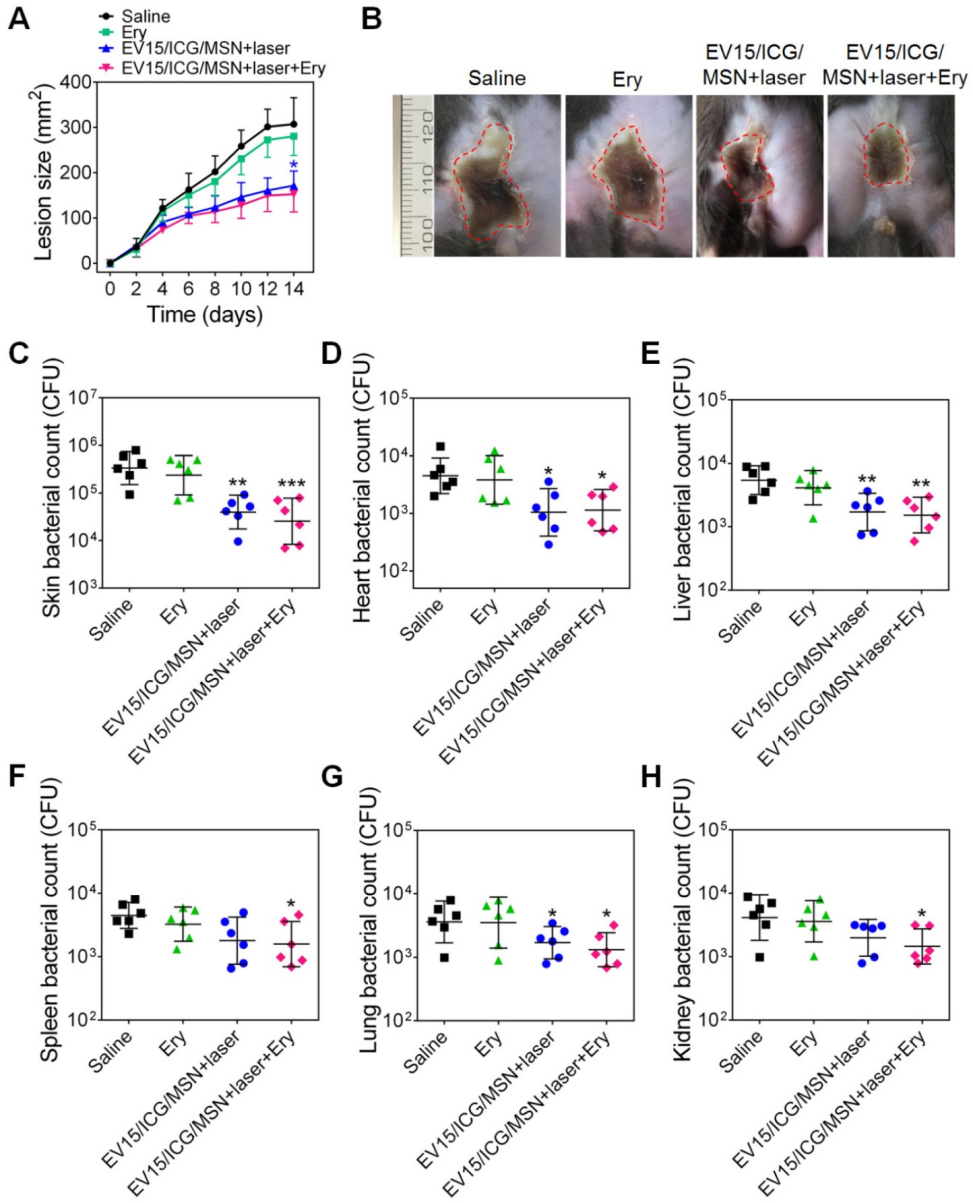
** EV-coated multi-antigenic nanovaccines-induced therapeutic protection against *S. aureus* BW15. (A)** Skin lesion sizes were monitored over the course of infection. (means ± SD; n = 6). **(B)** Images of skin lesions from different groups on day 14. **(C)** The affected skin and major organs, including the **(D)** heart, **(E)** liver, **(F)** spleen, **(G)** lung, and **(H)** kidney were collected and the bacterial burdens were enumerated. Bacterial enumeration data are presented as the geometric mean ± SD (n = 6). *P < 0.05, **P < 0.01, ***P < 0.005, vs the saline group.

To verify whether the nanovaccines could achieve therapeutic effects, the antibacterial activity of EV15/ICG/MSN was assessed. The effects of antibiotics and the combination of nanovaccines and antibiotics were investigated. The minimum inhibitory concentration (MIC) of erythromycin (Ery) against S. aureus BW15 was more than 256 μg/mL 28. The progression of skin lesion development in mice was monitored for 14 days. Figure 8A-B showed that an obvious skin infection was found in the saline group. By contrast, treatment with EV15/ICG/MSN with laser irradiation significantly inhibited lesion formation, indicating that it may be an effective therapy against the drug-resistant S. aureus strains. The antibiotic treatment had no significant effects on skin lesion inhibition compared to the saline group. The combination of nanovaccines with antibiotics did not significantly improve the reduction of the dermonecrotic area. The skin bacterial counts in Figure 8C confirmed these measurements along with the images of the lesion sizes.

The bacterial burdens in disseminated infections were further quantified (Figure 8D-H). Mice treated with EV15/ICG/MSN under laser irradiation or in combination with antibiotics showed significant reductions compared with the saline group (*P* < 0.05, *P* < 0.01). As shown by the lesion sizes, antibiotics alone had negligible effects in the number of bacteria isolated from major organs. These results suggest that the nanovaccines exhibit therapeutic efficacy in drug-resistant S. aureus-infected mice, which suggests that they may be translational candidates as an antimicrobial immunotherapy.

### Safety of EV-coated multi-antigenic nanovaccines

Lastly, we evaluated the potential side effects of EV-coated multi-antigenic nanovaccines. As shown in Figure S14A, a steady increase in the body weight of mice administrated with saline and EV/ICG/MSN under laser irradiation was observed. H&E staining results of the main organs (heart, liver, spleen, lung, and kidney) revealed no noticeable differences in any of the organs (Figure S14B). Moreover, administration of the EV/ICG/MSN with laser irradiation showed no significant changes in the levels of any of the measured blood biochemical indexes (Figure S14C). Overall, these findings support the safety of EV-coated multi-antigenic nanovaccines.

## Conclusions

Membrane vesicles coating has emerged as a promising approach which endows hybrid nanoparticles with long circulation times and disease-relevant targeting. The integration of biological properties of cell membranes and various functions of synthetic materials enable the nanoparticles with widespread applications, such as drug delivery, detection, imaging, detoxification and immune modulation 48. While red blood cell membrane, leukocyte membrane, platelet membrane, cancer cell membrane, and exosome have been used as the membrane source, there is some interest in using bacterium derived material 49-56. Bacterial double-layered membrane vesicles and the secreted EVs containing numerous bacterial components have gained much interest in vaccine development 57-59. In the present study, we have reported the generation of EV-coated multi-antigenic nanovaccines against drug-resistant* S. aureus* infection. We demonstrate the superior stability and rigidity to EVs, improved cellular uptake, enhanced proteasome activity, elevated costimulatory molecules, MHC-I and MHC-II, and upregulated cytokine expression that ensured DC maturation. Along with laser irradiation that caused effective endosomal escape, EV-coated multi-antigenic nanovaccines initiated cytosolic delivery and proteasome-dependent antigen presentation pathways for subsequent robust CD8^+^ T cell responses. The *in vivo* immune response indicated that the nanovaccines induced significantly improved CD8^+^ T cell responses while maintaining CD4^+^ T cell responses and humoral immunity. A mouse skin infection model was employed to evaluate drug-resistant* S. aureus*, and it was demonstrated that the nanovaccines could prevent and treat superficial infection, as well as systemic infection in these hosts, which suggests that these nanovaccines could ultimately be beneficial in preventing the complications associated with *S. aureus* infections. Overall, the nanovaccine-based multiple immune stimulatory molecules and adjustable antigen presentation pathways has a positive impact on the challenges of antibiotic-resistant bacteria. A current limitation of the developed nanovaccines is the inconvenience of laser irradiation. Further development of strategies for enhancing antigen cross-presentation will be taken into consideration, such as induction of osmotic swelling of endosomes, conjugation of endosome-disrupting peptides or side chains to nanoparticles, utilization of pH-responsive carriers in endosomes for promoting delivery of antigen into the cytoplasm. Furthermore, the antimicrobial effects should be improved by optimized design of formulation and dosage regimen.

## Supplementary Material

Supplementary figures and tables.

## Abbreviations

APCs: antigen presenting cells
BMDCs: bone marrow-derived dendritic cells
CTL: cytotoxic CD8^+^ T lymphocytes
TEM: transmission electron microscopy
PDI: polydispersity indexes
DPI: diphenylene iodonium
ELISA: enzyme-linked immunosorbent assay
Ery: erythromycin
EVs: extracellular vesicles
DCs: dendritic cells
ICG: indocyanine green
MFI: mean fluorescence intensity
F.I.: fluorescence intensity
IFN-γ: interferon-γ
LB: Luria broth
LNs: lymph nodes
CFU: colony forming units
MIC: minimum inhibitory concentration
*S. aureu*s: *Staphylococcus aureus*
MRSA: methicillin-resistant *S. aureus*
MSN: mesoporous silica nanoparticles
PBS: phosphate-buffered saline
*S. aureus*: *Staphylococcus aureus*
Th: T helper
