# Supplementary material for: Bacterial extracellular vesicle-coated multi-antigenic nanovaccines protect against drug-resistant Staphylococcus aureus infection by modulating antigen processing and presentation pathways
Source: Theranostics. 2020 May 30;10(16):7131–49. doi: 10.7150/thno.44564 (PMC7330855; doi:10.7150/thno.44564)
Supplement: Supplementary file 1 — Supplementary figures and tables. [file thnov10p7131s1.pdf]

Supplementary Information

Bacterial extracellular vesicle-coated multi-antigenic nanovaccines protect against drug-resistant *Staphylococcus aureus* infection by modulating antigen processing and presentation pathways

Gang Chen, Yanan Bai, Zhenzhen Li, Fei Wang, Xuelian Fan, Xin Zhou*

Institute of Comparative Medicine, College of Veterinary Medicine, Jiangsu Co-innovation Center for Prevention and Control of Important Animal Infectious Diseases and Zoonoses, Joint International Research Laboratory of Agriculture and Agri-Product Safety, the Ministry of Education of China, Yangzhou University, Yangzhou, 225009, China

*E-mail: zhou_xin@126.com (X. Zhou)

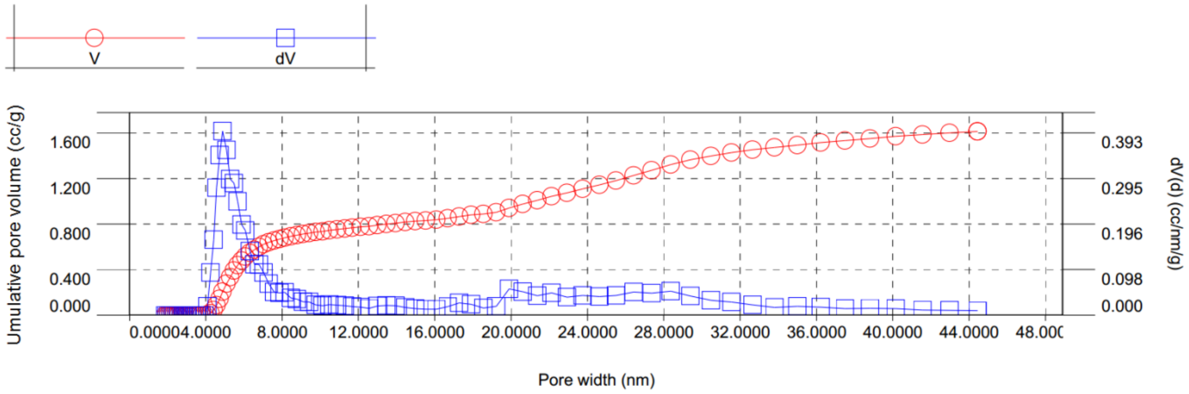

Figure S1. The pore width was determined by nitrogen adsorption assay.

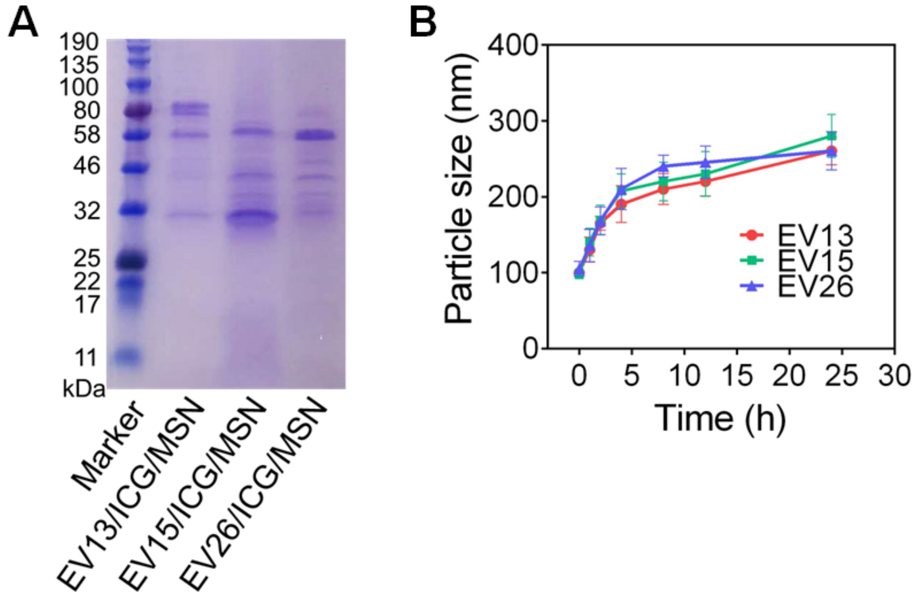

Figure S2. (A) SDS-PAGE analysis of EV-coated hybrid nanovaccines. (B) The size changes of EV13, EV15, and EV26 in PBS buffer. Data are presented as the means ± SD (n = 3).

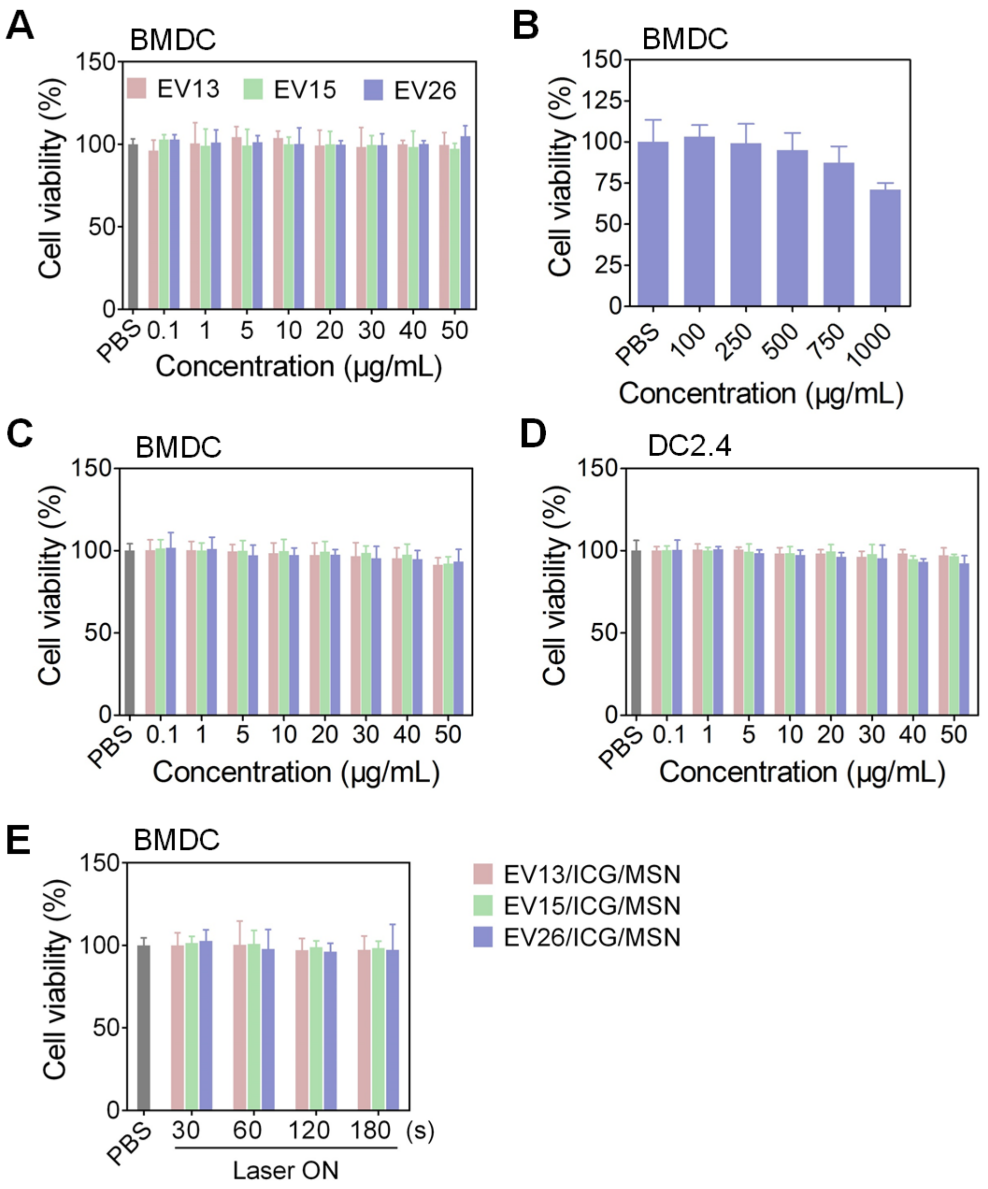

Figure S3. (A) Cell viability of BMDCs with EVs at different protein concentrations. (B) Cell viability of BMDCs with MSN at different concentrations. Cell viability of (C) BMDCs and (D) DC2.4 cells with EV-coated nanovaccines at different protein concentrations. (E) Cell viability of BMDCs treated with EV-coated hybrid nanovaccines exposed to laser irradiation for 30-180 s. Data are presented as the means ± SD (n = 3).

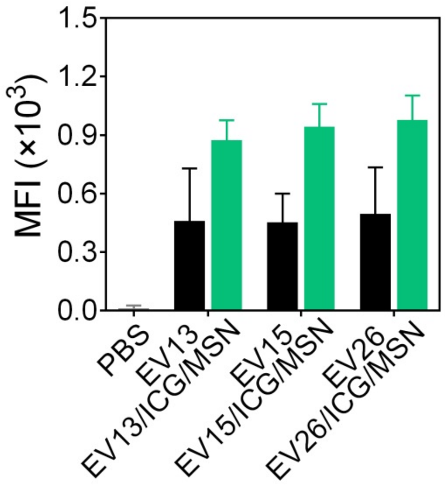

Figure S4. Cell uptake by BMDCs determined by flow cytometry at 4 h post-incubation with EVs and EV-coated hybrid nanovaccines. Data are presented as the means ± SD (n = 3).

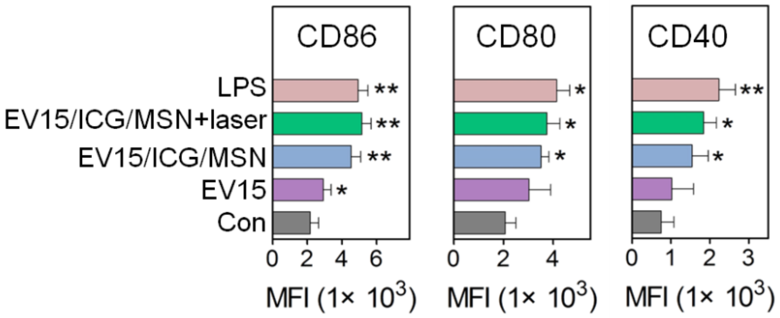

Figure S5. Mean fluorescence intensity (MFI) of CD86, CD80 and CD40 expression by BMDCs was determined by flow cytometry. Data are presented as the means ± SD (n = 3). $^*P < 0.05$, $^{**}P < 0.01$, vs the control group.

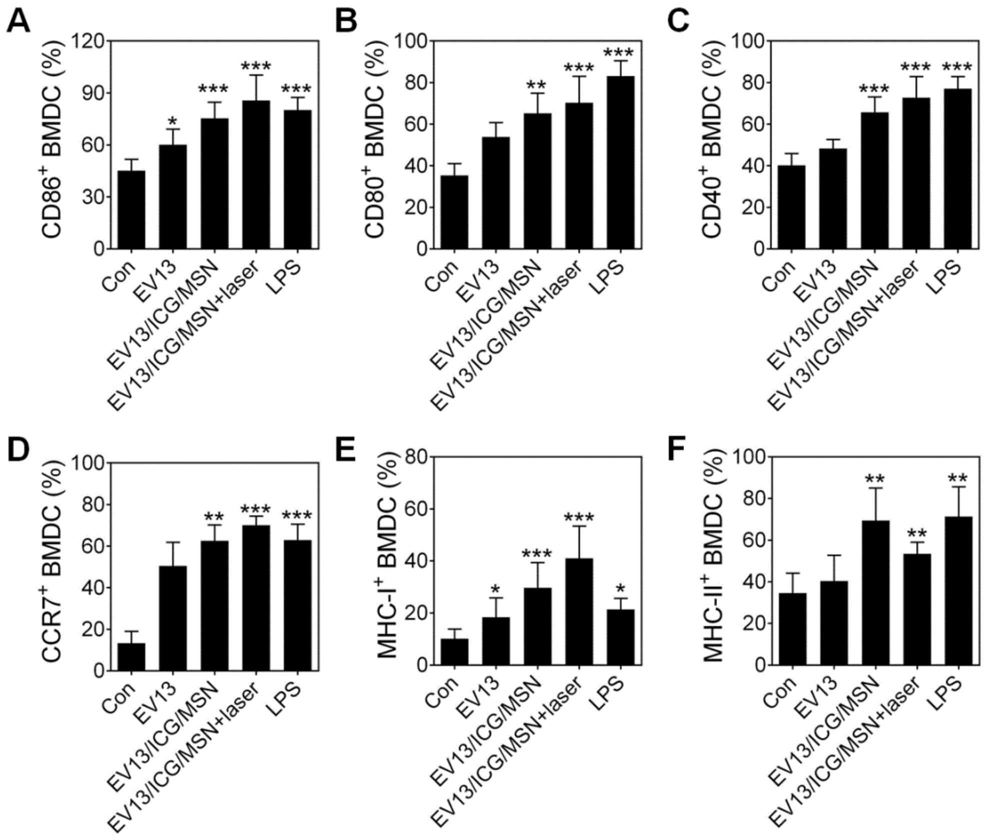

Figure S6. (A)-(F) BMDCs were analysed for expression of CD86, CD80, CD40, CCR7, MHC-I and MHC-II by flow cytometry after stimulation with EV13, EV13/ICG/MSN, EV13/ICG/MSN+laser, and LPS. Data are presented as the means ± SD (n = 3). $^{*}P < 0.05$, $^{**}P < 0.01$, $^{***}P < 0.005$, vs the control group.

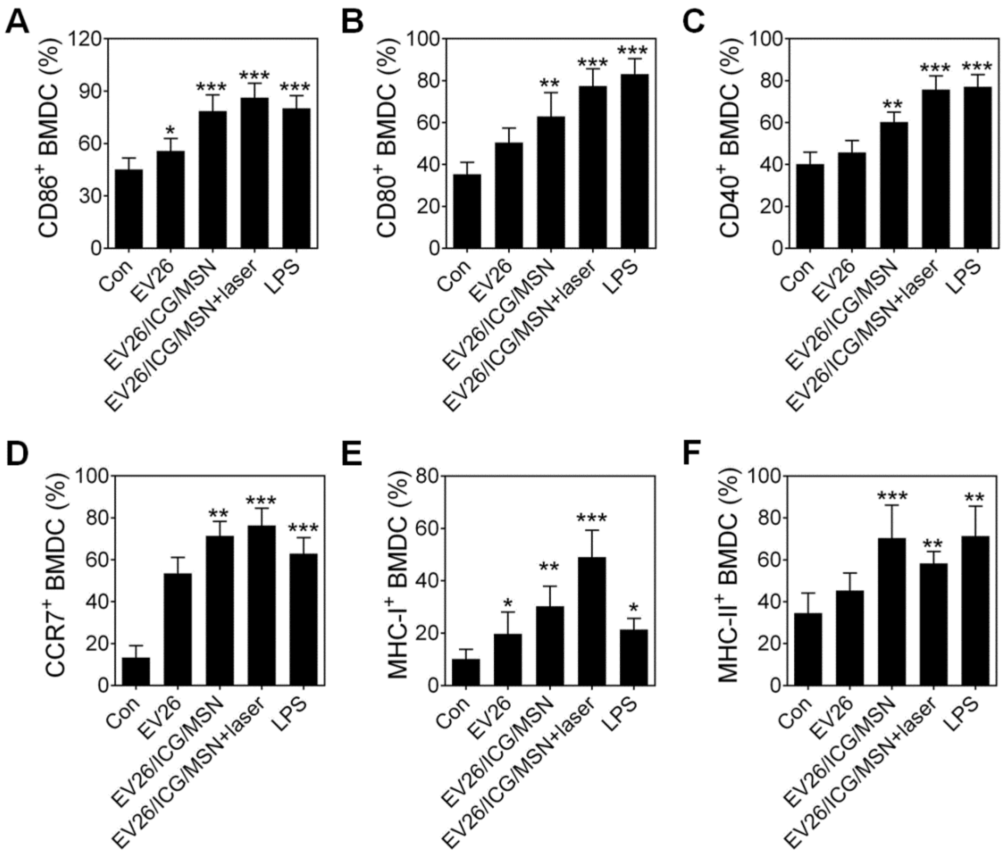

Figure S7. (A)-(F) BMDCs were analysed for expression of CD86, CD80, CD40, CCR7, MHC-I and MHC-II by flow cytometry after stimulation with EV26, EV26/ICG/MSN, EV26/ICG/MSN+laser, and LPS. Data are presented as the means ± SD (n = 3). $^{*}P < 0.05$, $^{**}P < 0.01$, $^{***}P < 0.005$, vs the control group.

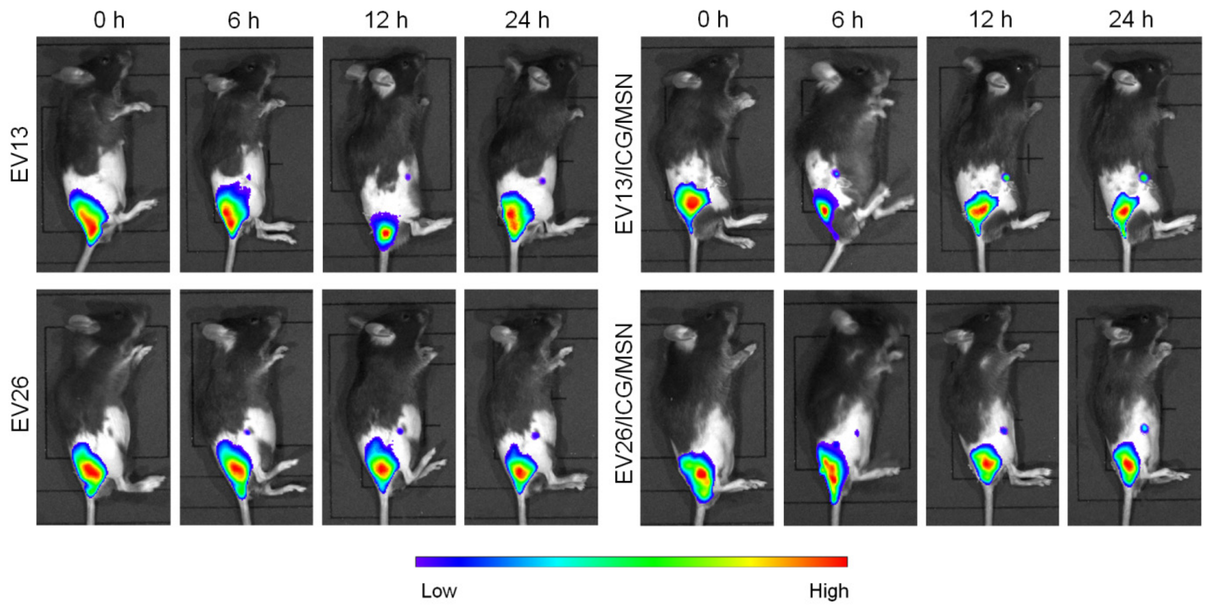

Figure S8. *In vivo* fluorescent images of EV13, EV13/ICG/MSN, EV26, and EV26/ICG/MSN at different time points.

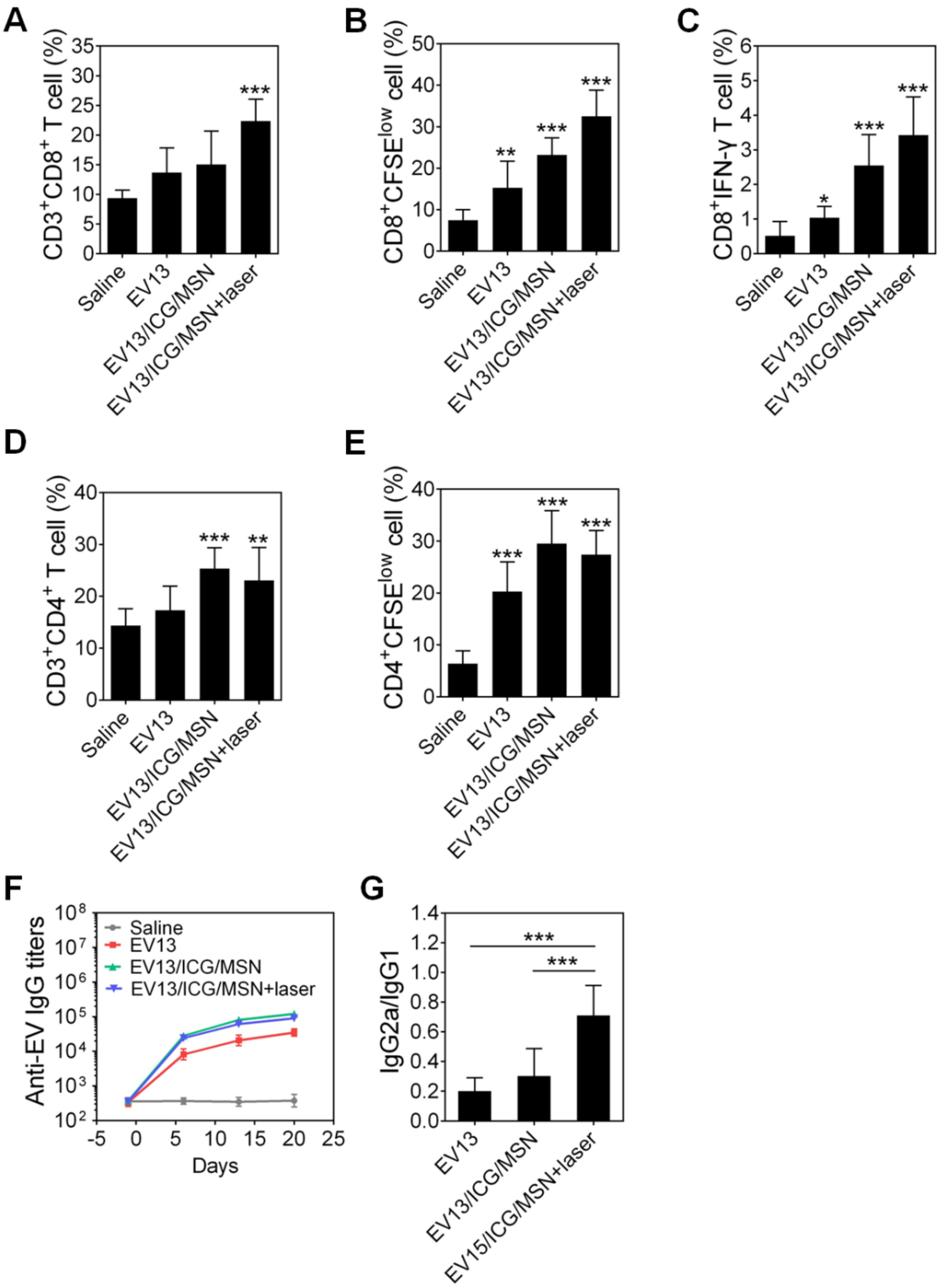

Figure S9. (A) The proportion of $CD3^{+}CD8^{+}$ T cells in splenocytes was determined by flow cytometry after stimulation with EV13, EV13/ICG/MSN, and EV13/ICG/MSN+laser. (B) The proliferation of $CD8^{+}$ T cells was assessed by CFSE dilution. (C) The proportion of IFN-γ-producing $CD8^{+}$ T cells was determined by flow cytometry. (D) The proportion of $CD3^{+}CD4^{+}$ T cells in splenocytes was determined by flow cytometry. (E) The proliferation of $CD4^{+}$ T cells was assessed by CFSE dilution. (F) Time course of EV-specific IgG titres. (G) The IgG2a/IgG1 ratio was measured on day 21 in sera from immunized mice. Data are presented as the means ± SD (n = 6). $^{*}P < 0.05$, $^{**}P < 0.01$, $^{***}P < 0.005$, vs the saline or indicated groups.

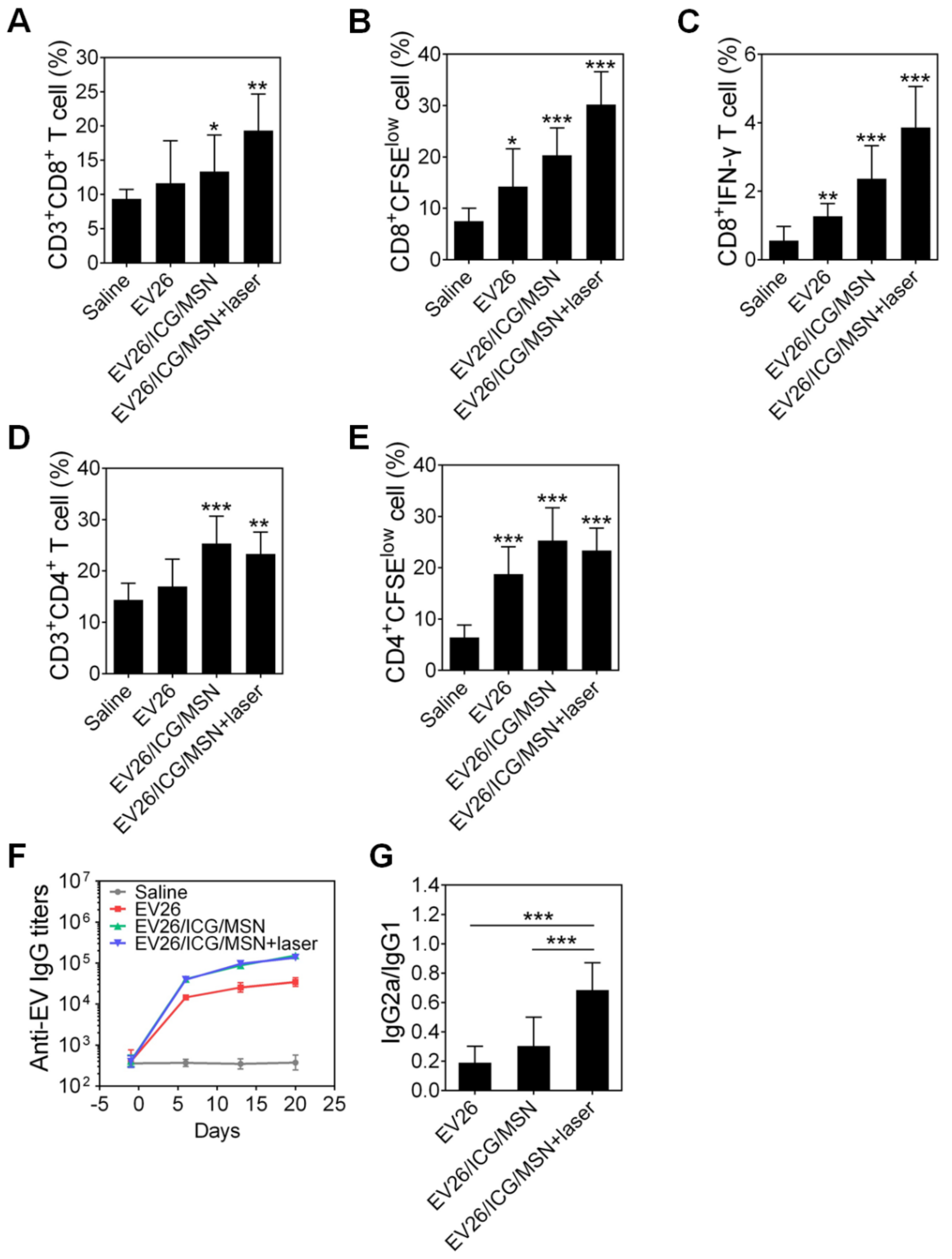

Figure S10. (A) The proportion of CD3$^+$CD8$^+$ T cells in splenocytes was determined by flow cytometry after stimulation with EV26, EV26/ICG/MSN, and EV26/ICG/MSN+laser. (B) The proliferation of CD8$^+$ T cells was assessed by CFSE dilution. (C) The proportion of IFN-γ-producing CD8$^+$ T cells was determined by flow cytometry. (D) The proportion of CD3$^+$CD4$^+$ T cells in splenocytes was determined by flow cytometry. (E) The proliferation of CD4$^+$ T cells was assessed by CFSE dilution. (F) Time course of EV-specific IgG titres. (G) The IgG2a/IgG1 ratio was measured on day 21 in sera from immunized mice. Data are presented as the means ± SD (n = 6). $^*P < 0.05$, $^{**}P < 0.01$, $^{***}P < 0.005$, vs the saline or indicated groups.

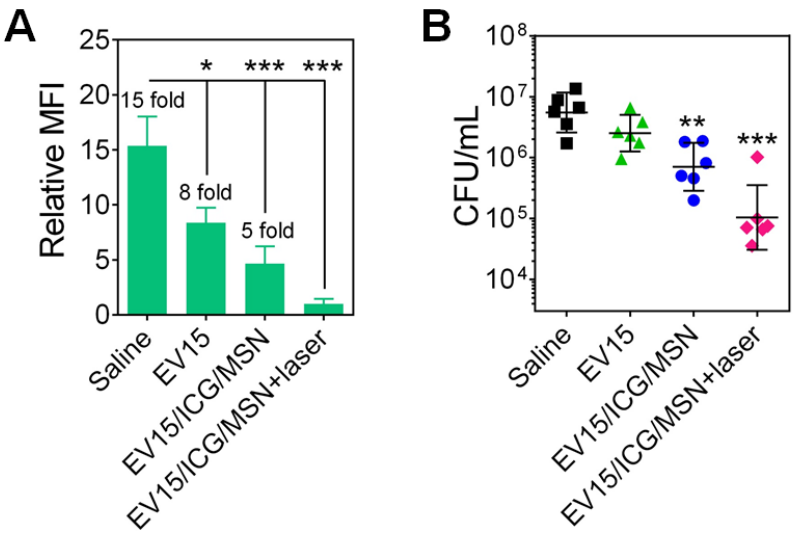

Figure S11. (A) The proliferation of $CD8^+$ T cells was assessed by CFSE dilution after restimulation (means ± SD; n = 6). (B) Antimicrobial assays against intracellular *S. aureus* within macrophage cells (geometric mean ± SD; n = 6). $^{*}P < 0.05$, $^{**}P < 0.01$, $^{***}P < 0.001$, vs the saline group.

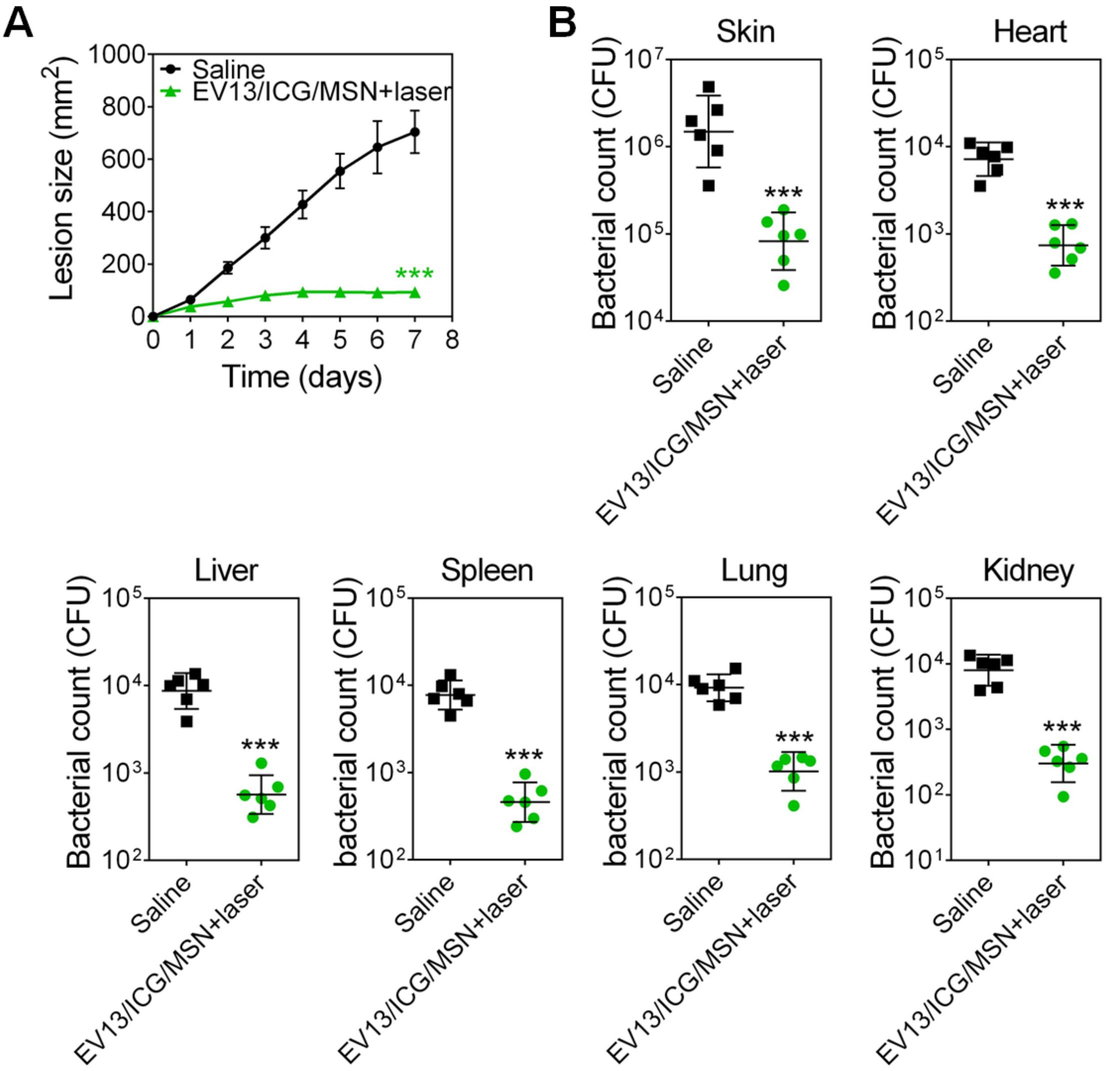

Figure S12. (A) Skin lesions were monitored in mice over the course of infection with *S. aureus* S29213 (means ± SD; n = 6). (B) On day 7 post-infection, the infected skin and major organs, including the heart, liver, spleen, lung, and kidney were collected and the bacterial burdens were enumerated (geometric mean ± SD; n = 6). $^{**}P < 0.01$, $^{***}P < 0.005$, vs the saline group.

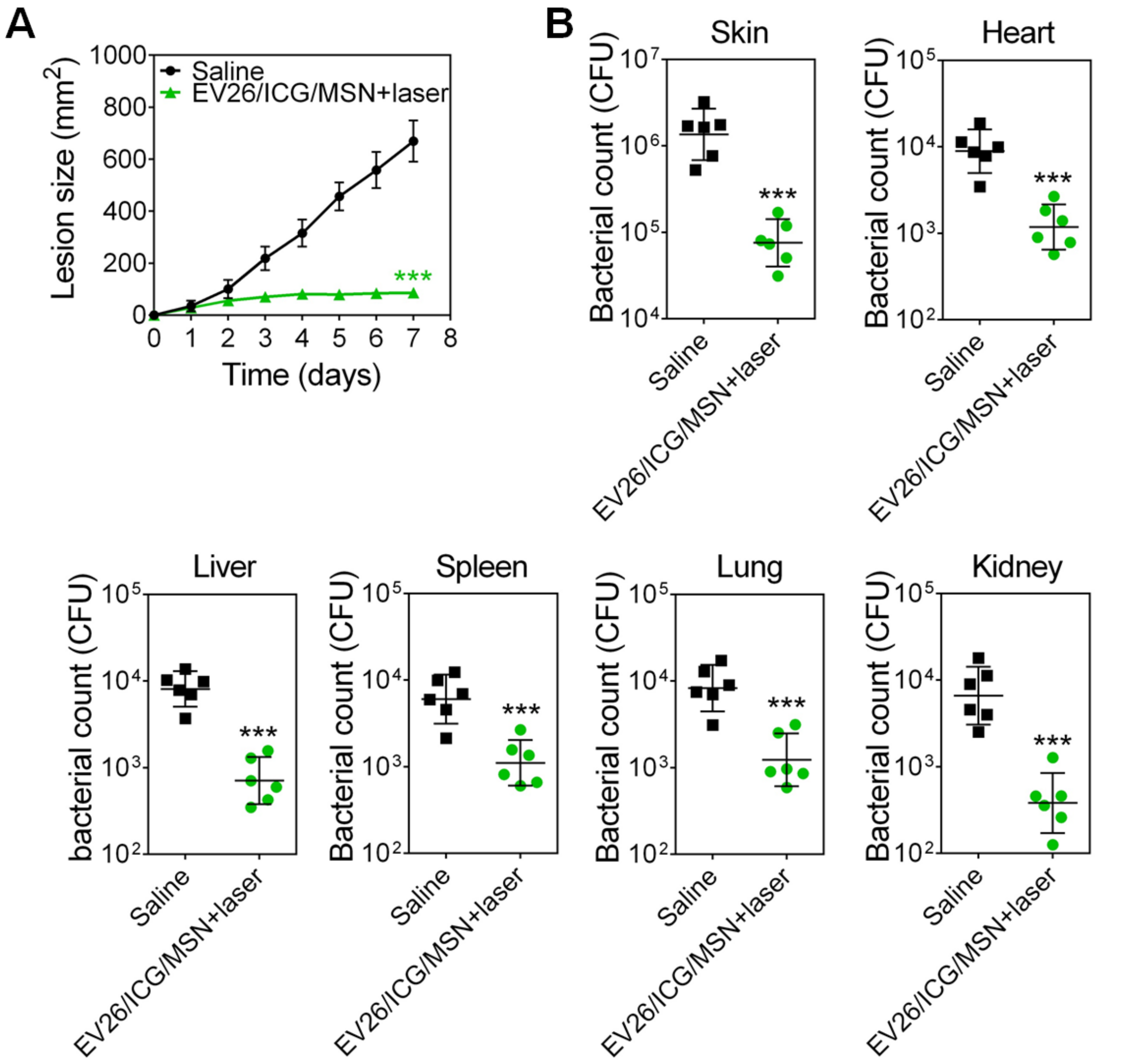

Figure S13. (A) Skin lesions were monitored over the course of infection in mice challenged with *S. aureus* BWMR26 (means ± SD; n = 6). (B) On day 7 post-infection, the affected skin and major organs, including the heart, liver, spleen, lung, and kidney were collected and the bacterial burdens were enumerated (geometric mean ± SD; n = 6). $^{**}P < 0.01$, $^{***}P < 0.005$, vs the saline group.

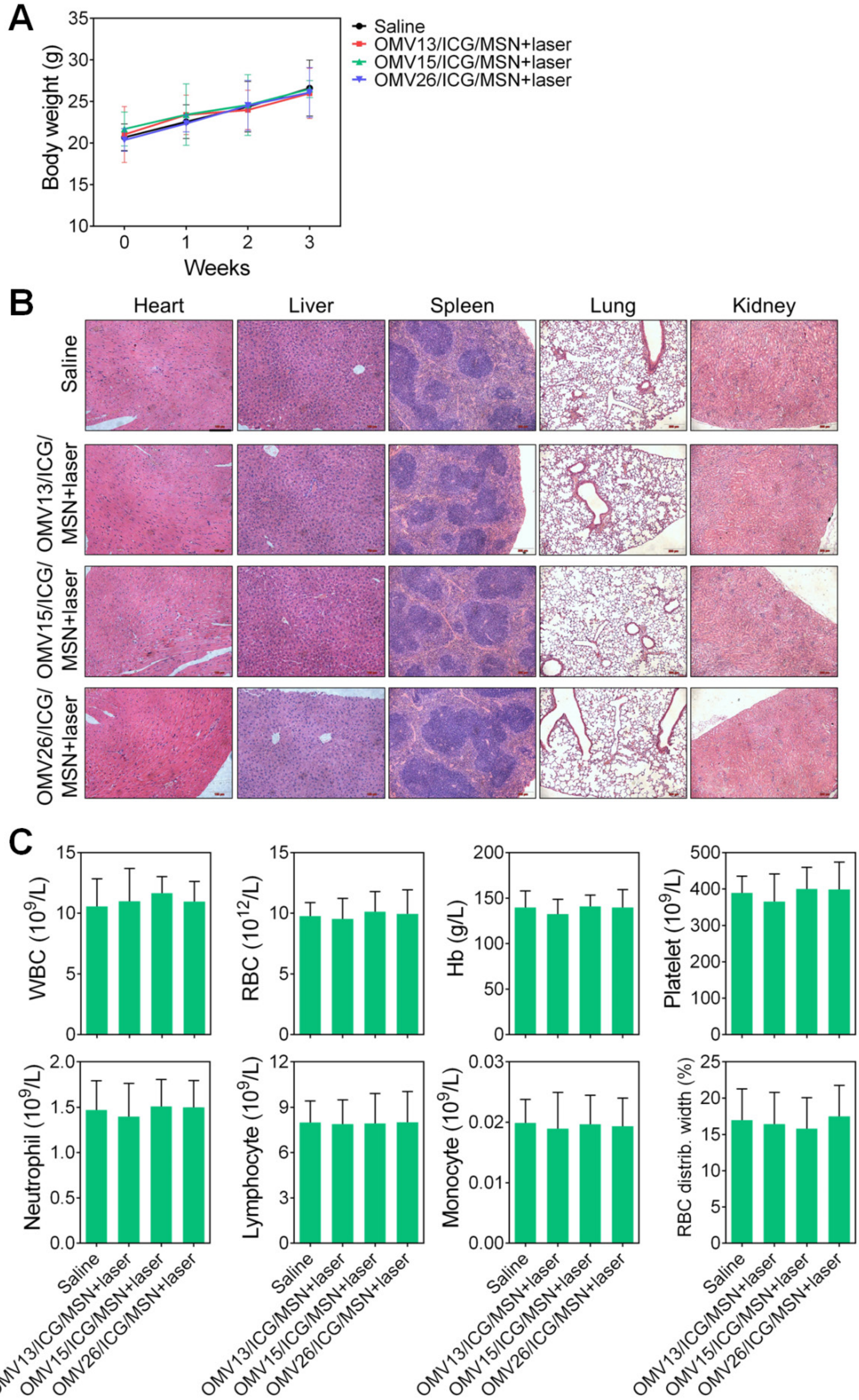

Figure S14. (A) Body weight changes. (B) H&E stained images of the main organs. (C) Blood biochemical indexes of mice administrated with EV/ICG/MSN+laser or saline (mean ± SD; n=6).
